# The IRAK-M death domain: a tale of three surfaces

**DOI:** 10.3389/fmolb.2023.1265455

**Published:** 2024-01-10

**Authors:** Berke Gürkan, Hessel Poelman, Liza Pereverzeva, Danielle Kruijswijk, Alex F. de Vos, Anouk G. Groenen, Edgar E. Nollet, Kanin Wichapong, Esther Lutgens, Tom van der Poll, Jiangfeng Du, W. Joost Wiersinga, Gerry A. F. Nicolaes, Cornelis van ‘t Veer

**Affiliations:** ^1^ Center of Experimental and Molecular Medicine, Amsterdam UMC Location University of Amsterdam, Amsterdam, Netherlands; ^2^ Amsterdam Infection and Immunity Institute, Amsterdam UMC Location University of Amsterdam, Amsterdam, Netherlands; ^3^ Department of Biochemistry, Cardiovascular Research Institute Maastricht, Maastricht University, Maastricht, Netherlands; ^4^ Medical Biochemistry, Amsterdam UMC Location University of Amsterdam, Amsterdam, Netherlands; ^5^ Division of Infectious Diseases, Amsterdam UMC Location University of Amsterdam, Amsterdam, Netherlands; ^6^ School of Life Sciences, Zhengzhou University, Zhengzhou, China

**Keywords:** IRAK-M, IRAK-3, myddosome, death domain, Toll-like receptors, inflammation

## Abstract

The anti-inflammatory interleukin-1 receptor associated kinase-M (IRAK-M) is a negative regulator of MyD88/IRAK-4/IRAK-1 signaling. However, IRAK-M has also been reported to activate NF-κB through the MyD88/IRAK-4/IRAK-M myddosome in a MEKK-3 dependent manner. Here we provide support that IRAK-M uses three surfaces of its Death Domain (DD) to activate NF-κB downstream of MyD88/IRAK-4/IRAK-M. Surface 1, with central residue Trp74, binds to MyD88/IRAK-4. Surface 2, with central Lys60, associates with other IRAK-M DDs to form an IRAK-M homotetramer under the MyD88/IRAK-4 scaffold. Surface 3; with central residue Arg97 is located on the opposite side of Trp74 in the IRAK-M DD tetramer, lacks any interaction points with the MyD88/IRAK-4 complex. Although the IRAK-M DD residue Arg97 is not directly involved in the association with MyD88/IRAK-4, Arg97 was responsible for 50% of the NF-κB activation though the MyD88/IRAK-4/IRAK-M myddosome. Arg97 was also found to be pivotal for IRAK-M’s interaction with IRAK-1, and important for IRAK-M’s interaction with TRAF6. Residue Arg97 was responsible for 50% of the NF-κB generated by MyD88/IRAK-4/IRAK-M myddosome in IRAK-1/MEKK3 double knockout cells. By structural modeling we found that the IRAK-M tetramer surface around Arg97 has excellent properties that allow formation of an IRAK-M homo-octamer. This model explains why mutation of Arg97 results in an IRAK-M molecule with increased inhibitory properties: it still binds to myddosome, competing with myddosome IRAK-1 binding, while resulting in less NF-κB formation. The findings further identify the structure-function properties of IRAK-M, which is a potential therapeutic target in inflammatory disease.

## 1 Introduction

MyD88 is an intracellular signaling molecule involved in the host response to microbial infections, viral and bacterial PAMPs and endogenous danger signals provided by Toll-like receptors (TLRs) and IL-1, IL-18 and IL-33 receptors (Wesche et al., 1997; Yamamoto et al., 2003; Takeuchi and Akira, 2010). MyD88 consists of a C-terminal Toll/Interleukin-1 receptor (TIR) domain and an N-terminal Death Domain (DD). Receptor activation induces TIR dependent dimerization of MyD88 (Medzhitov et al., 1997; Muzio et al., 1997; Jiang et al., 2005) bringing the first two MyD88 DDs together. These form the scaffold for a new layer of 4 MyD88 molecules by DD-DD interactions forming a left-handed helix structure (Lin et al., 2010; Ve et al., 2017). This MyD88 tetramer is the lattice for association of four DDs of Interleukin-1 Receptor-Associated Kinase (IRAK)-4 adding a second layer to the helix. Consecutively, this latter IRAK-4 layer recruits IRAK-1 or IRAK-2 in a similar DD dependent manner to form a third DD tetramer layer to generate so called myddosomes (MyD88/IRAK-4/IRAK-1 or MyD88/IRAK-4/IRAK-2) in which IRAK-1 and IRAK2 can be activated through phosphorylation by IRAK-4 (Li et al., 2002; Suzuki et al., 2002; Pennini et al., 2013). IRAK-1 and IRAK-2 myddosomes recruit and oligomerize the E3 ligase TRAF6 which results in TAK-1 dependent activation of nuclear factor-κB (NF-κB) and MAP kinases (Muzio et al., 1997; Li et al., 2001; Wang et al., 2001; Ye et al., 2002; Lamothe et al., 2008). These initiate the transcription and translation of inflammatory cytokines, adhesion molecules and expression of anti-microbial factors to deal with the recognized microbe or aberrant host-related factors/molecules (Takeuchi and Akira, 2010). MyD88 and IRAK-4 deficiency results in increased susceptibility to bacterial infections in mice and humans [see Pennini et al. (2013) and references in there].

IRAK-M (IRAK-3), a homolog of IRAK-1, 2 and 4 that lacks apparent kinase activity, regulates the MyD88-IRAK pathway in monocytes/macrophages and lung epithelial cells and has an overall inhibitory effect on TLR mediated cytokine release *in vitro* (Wesche et al., 1999; Kobayashi et al., 2002; Zhou et al., 2013). Human IRAK-M has been reported to interfere with the transient IRAK-1/TRAF6 interaction upon IL-1 and Toll-like receptor stimulation, and to stabilize IRAK-1 and IRAK-4 myddosome complexes, presumably by inhibition of IRAK-4/IRAK-1 kinase activity (Kobayashi et al., 2002). In the murine system IRAK-M has been shown to inhibit cytokine production by a pathway which involves MyD88/IRAK-4/IRAK-M/MEKK3 dependent NF-κB activation that specifically induces the expression of known inflammatory inhibitors such as SOCS-1, A20, IκBα and SHIP1 (Kobayashi et al., 2002). Moreover, IRAK-M may downregulate IRAK-2 dependent translation of proinflammatory transcripts (Kobayashi et al., 2002). Mutations in IRAK-M are associated with early onset asthma and IRAK-M deficiency affects IL-33 induced asthmatic responses in mice (Balaci et al., 2007; Nechama et al., 2018). While IRAK-1 is downregulated, IRAK-M is upregulated by inflammatory reactions (van ‘t Veer et al., 2007), and this rebalance of the IRAK system appears to be causal for the observed susceptibility to nosocomial infections after a first inflammatory insult (Deng et al., 2006). IRAK-M deficient mice exhibit an increased inflammatory response and display increased survival in bacterial pneumonia models (Kobayashi et al., 2002; Deng et al., 2006; Hoogerwerf et al., 2012; van der Windt et al., 2012), and an improved host response in tumor models (Xie et al., 2007) and bone marrow transplantation (Hubbard et al., 2010). Therefore, targeting of IRAK-M has therapeutic potential in infectious and oncologic disease. While the overall anti-inflammatory action of IRAK-M is well established in murine macrophages (Kobayashi et al., 2002; Deng et al., 2006; Zhou et al., 2013), it remains poorly understood how human IRAK-M affects the human immune system.

**FIGURE 1:**
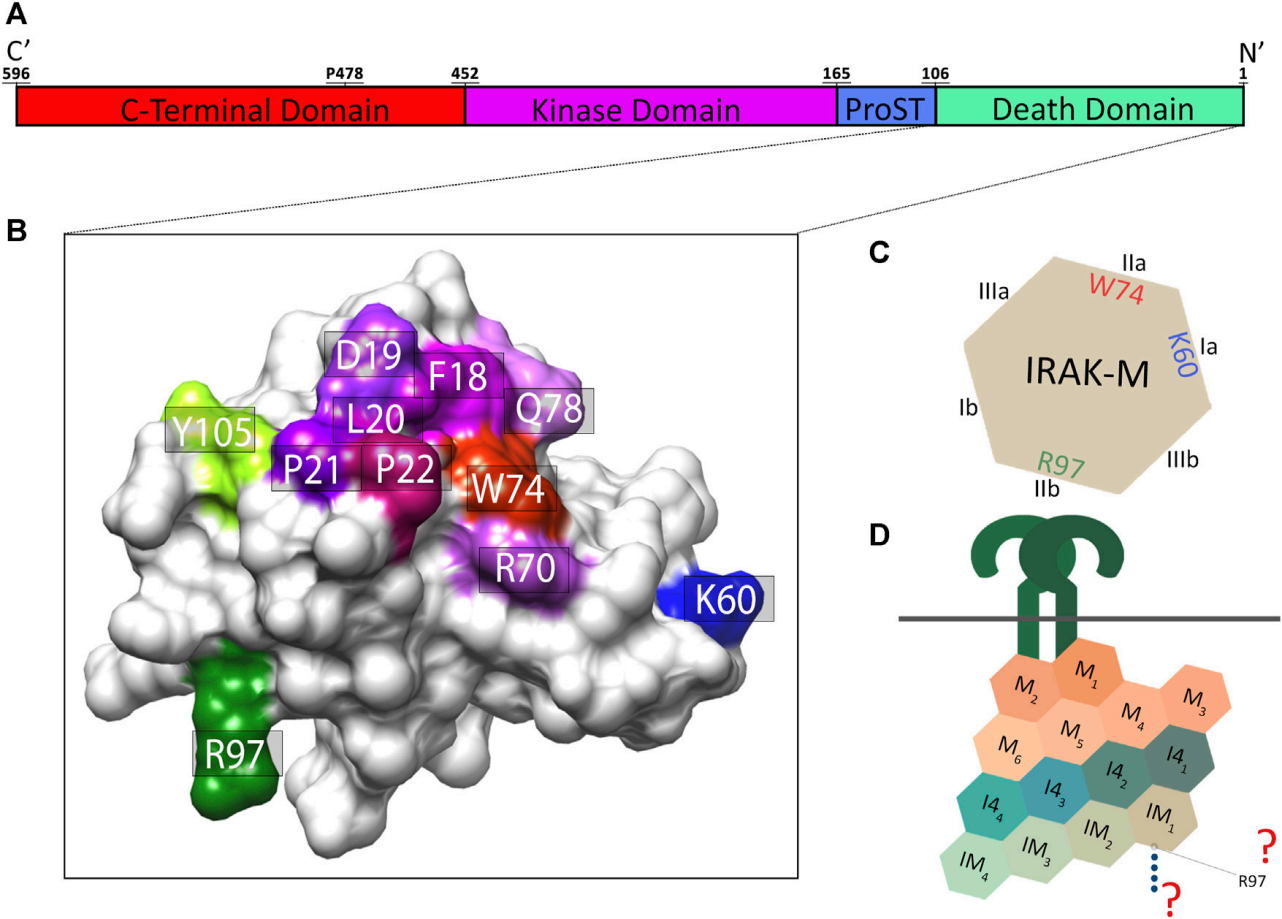
Schematic representations of IRAK-M Death Domain and its potential interactions in the myddosome. **(A)** IRAK-M secondary structure. IRAK-M contains 4 distinct domains: Death Domain, ProST Domain, Kinase Domain and C-Terminal Domain. Residue Pro478 on the C-terminal domain is part of the TRAF-6 binding motif. Indicated are the borders of the domains, the secondary structure is shown from C to N terminal to fit the best view orientation with **(B–D)**. **(B)** Predicted IRAK-M Death Domain structure with identified functional residues indicated, based on the homology model by Poelman et al. (2022). **(C)** Schematic representation of IRAK-M Death Domain interaction surfaces as described by Lin et al. (2010). **(D)** Schematic representation of IRAK-M/Myddosome interaction. M = MyD88, I4 = IRAK-4, IM = IRAK-M.

IRAK-M is structurally similar to the other IRAK family members (IRAK-1, IRAK-2, IRAK-4) with a N-terminal DD, a middle Kinase Domain (KD) and an unstructured C-Terminal Domain (CTD) with a single TRAF6 binding motif at Pro478 (Wesche et al., 1999; Flannery and Bowie, 2010) (Figure 1A). Apart from interaction of IRAK-M with MyD88/IRAK-4 oligomers, which is facilitated by the IRAK-M DD surface pivoted by Trp74 (Zhou et al., 2013), little is known about the structural requirements of IRAK-M to interact with its other binding partners. Our previous study (Du et al., 2014) showed that another predicted protein interactive region on the IRAK-M DD, pivoted by residue Arg97, also impacts the function of IRAK-M (Figure 1B). Based on a model of the IRAK-M DD tetramer (Du et al., 2014), we hypothesized that the Arg97 residue of the IRAK-M DD is on the opposite face of the IRAK-M tetramer than the Trp74 face that binds IRAK-4 in the MyD88/IRAK-4 complex (Kobayashi et al., 2002; Du et al., 2014). In this model Trp74 is part of a type IIa interaction surface, as defined by Lin et al. (2010) for the MyD88/IRAK DD interactions, and Arg97 would be part of the exact opposite side of the Death Domain pivoting a type IIb interaction surface (Figure 1C). Remarkably, mutation of IRAK-M residue Arg97 resulted in a mutant with diminished NF-κB activating capacity, less inhibitory function on TLR2/4 mediated cytokine release in the human monocyte cell line THP-1, but unexpectedly in a more potent inhibitor of TLR5 mediated IL-8 release in the human bronchial lung epithelial cell line H292 (Du et al., 2014).

Here we show by immunoprecipitation that residue Arg97 of IRAK-M is not involved in the interaction with the MyD88/IRAK-4 complex but important in IRAK-1 and TRAF6 binding. Moreover, our present findings indicate that the Arg97 side of IRAK-M is involved in MyD88/IRAK-4/IRAK-M myddosome activity towards NF-κB in a MEKK3 independent manner. Our results suggest that IRAK-M can form homo-oligomers larger than tetramers either in solution or under the MyD88/IRAK-4/IRAK-M myddosome. Structure modeling indicates that this entails the recruitment of additional IRAK-M subunits into the DD tetramer via its exposed Arg97, thereby extending the left-handed DD helix.

## 2 Results

### 2.1 IRAK-M/IRAK-4 interaction in the myddosome is independent of the Arg97 DD face of IRAK-M

**FIGURE 2:**
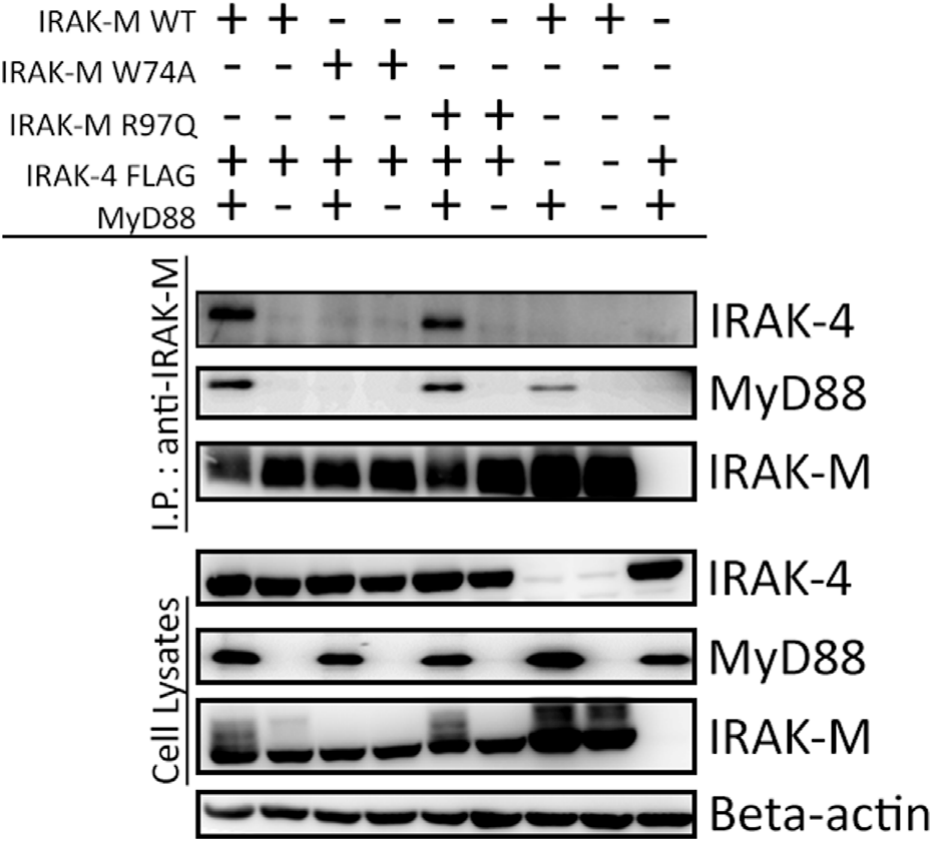
MyD88/IRAK-4/IRAK-M interaction is independent of IRAK-M residue Arg97. IRAK-M-WT, IRAK-M-W74A or IRAK-M-R97Q mutants were co-expressed with IRAK-4-FLAG and MyD88-HA in 293T cells and subsequently immunoprecipitated. Both IRAK-M WT and IRAK-M R97Q show a stable interaction with the myddosome scaffold formed by the overexpression of MyD88 and IRAK-4. W74A mutation prevents this interaction. Immunoprecipitation was performed using anti-IRAK-M (1F6) antibody. IRAK-4 and MyD88 were detected using anti-FLAG and anti-HA antibodies respectively. IRAK-M was detected using anti-IRAK-M (4369). β-actin is used as loading control. Data is representative of 3 independent experiments.

**FIGURE 3:**
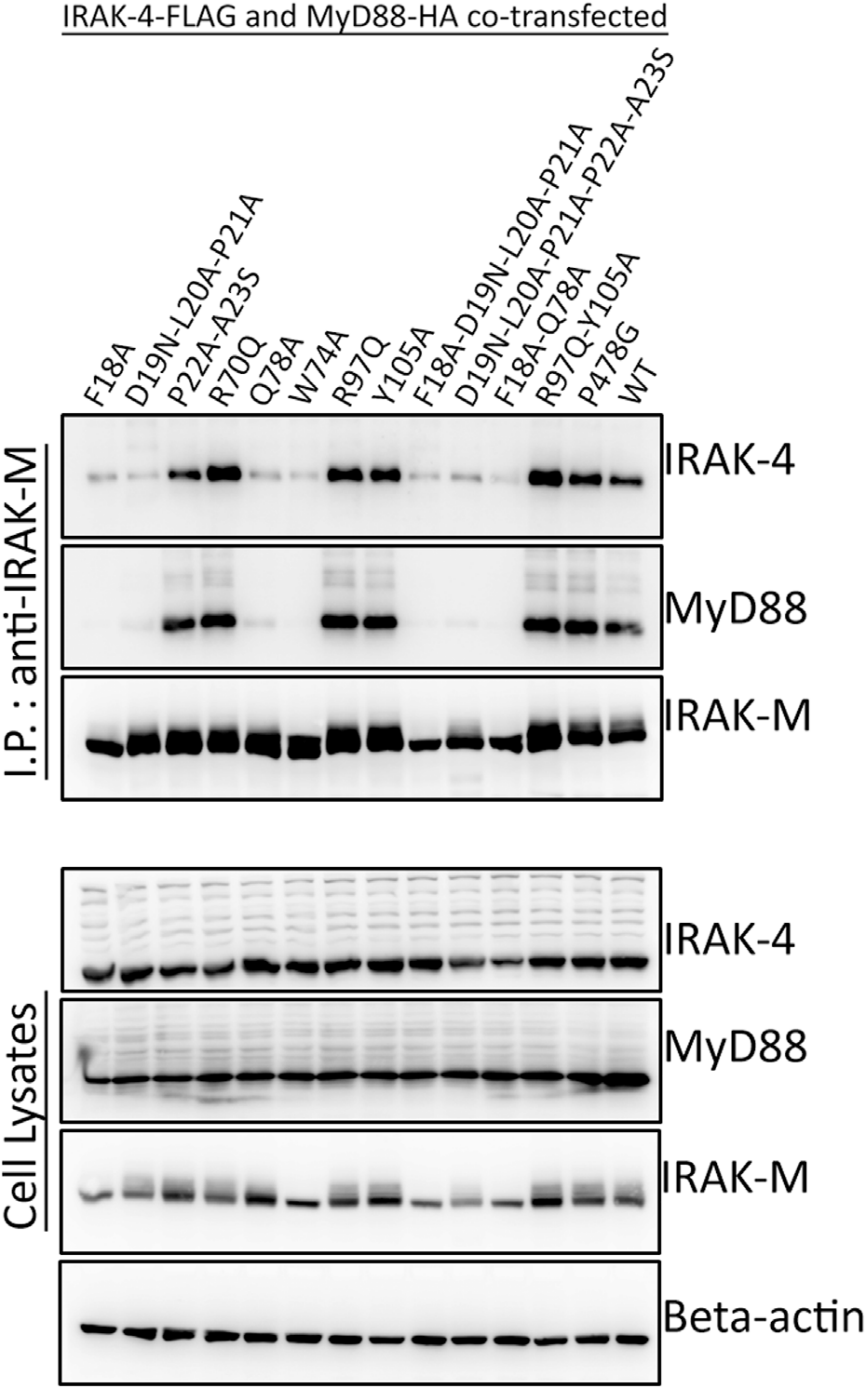
MyD88/IRAK-4/IRAK-M interaction depends on residues surrounding IRAK-M residue Trp74. IRAK-M-WT or indicated IRAK-M mutants were coexpressed with IRAK-4-FLAG and MyD88-HA in 293T cells and subsequently immunoprecipitated. IRAK-M interaction with the myddosome is hampered when the residues surrounding Trp74 are mutated. IRAK-M R97Q mutation does not affect this interaction. Immunoprecipitation was performed using anti-IRAK-M (1F6) antibody. IRAK-4 and MyD88 were detected using anti-FLAG and anti-HA antibodies respectively. IRAK-M was detected using anti-IRAK-M (4369). β-actin is used as loading control. Data is representative of 3 independent experiments.

To determine whether the binding surface on IRAK-M provided by Arg97 is involved in direct binding of IRAK-4 or to the MyD88/IRAK-4 complex, we co-expressed WT IRAK-M and R97Q mutant with IRAK-4 in 293T cells in the presence and absence of MyD88. Upon immunoprecipitation of IRAK-M, we were unable to observe an interaction between IRAK-M and IRAK-4 in the absence of exogenous MyD88 overexpression (Figure 2), indicating that IRAK-M and IRAK-4 do not form stable hetero-oligomers in the absence of MyD88 oligomers, even at the high concentrations attained by the overexpression of both. In contrast, in the presence of a stable MyD88/IRAK-4 myddosome, driven by co-expression of MyD88, stable MyD88/IRAK-4/IRAK-M myddosome complexes formed, as judged by co-immunoprecipitation of both MyD88 and IRAK-4 with WT IRAK-M (Figure 2). Under these conditions, IRAK-M residue Trp74 and surrounding residues Phe18, Asp19-Pro21 and Gln78 are pivotal for MyD88/IRAK-4/IRAK-M complexation (Figures 2, 3). Although residues Pro22/Ala23 are predicted to be protein interactive and presumably located at the Trp74 side of an IRAK-M DD tetramer (Du et al., 2014) these residues did not appear to contribute to MyD88/IRAK-4/IRAK-M myddosome formation (Figure 3). Complexation of MyD88/IRAK-4/IRAK-M occurred completely independent of IRAK-M residue Arg97 and Tyr105 (Figures 2, 3), in agreement with the predicted location of Arg97 on the other side of IRAK-M tetramer than the one that interacts with the MyD88/IRAK-4 complex via Trp74 (18, 27, Figures 1C, D). An IRAK-M mutant that lacks the TRAF6 binding motif in its C-terminal domain (P478G), associated to MyD88/IRAK-4 similar as wild type IRAK-M (Figure 3), indicating a lack of contribution of TRAF6 binding of IRAK-M to this interaction. Taken together these findings confirm the binding of IRAK-M to the MyD88/IRAK-4 complex via the Trp74 interactive surface. This interaction does not involve residue Arg97 of IRAK-M.

### 2.2 Stable IRAK-M/IRAK-1 complexes depend on residue Trp74 as well as Arg97 of IRAK-M

**FIGURE 4:**
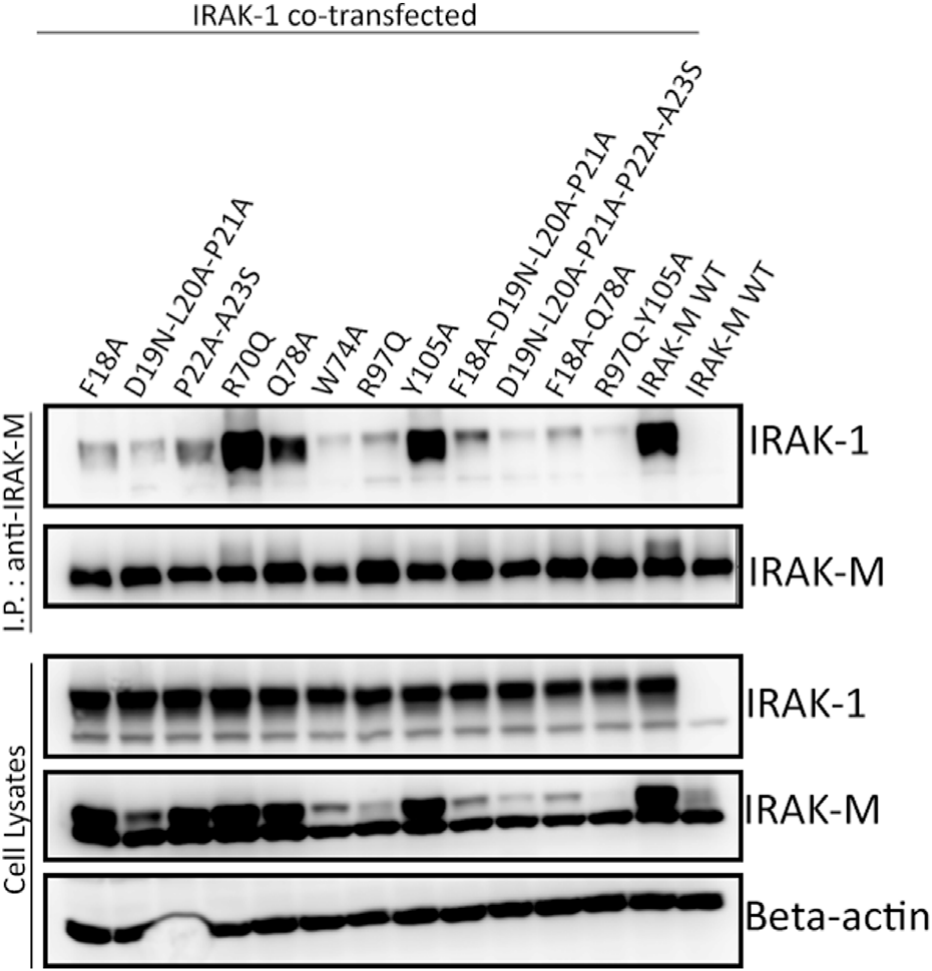
IRAK-1/IRAK-M interaction depends on IRAK-M residues Trp74 and Arg97. IRAK-M-WT or indicated IRAK-M mutants were coexpressed with IRAK-1 in 293T cells and subsequently immunoprecipitated. IRAK-M WT can stably interact with IRAK-1, whereas both W74A and R97Q mutations hamper this interaction. Immunoprecipitation was performed using anti-IRAK-M (1F6) antibody. IRAK-1 and IRAK-M were detected using anti-IRAK-1 and anti-IRAK-M (4369) antibodies. β-actin is used as loading control. Data is representative of 3 independent experiments.

In contrast to IRAK-4, IRAK-1 interacted with IRAK-M upon co-expression in a myddosome and IRAK-4 independent fashion in 293T cells (Figure 4; Supplementary Figure S1). This interaction is dependent on IRAK-M residues Trp74 as well as Arg97 (Figure 4, lanes 6 and 7) suggesting that both sides of the IRAK-M tetramer associate with IRAK-1. The residues directly adjacent to Trp74 (Phe18, Asp19-Pro21 and Gln78) are also involved in the interaction with IRAK-1, similar to the importance of these residues for the IRAK-M/MyD88/IRAK-4 interaction. Interestingly, while IRAK-M residues Pro22 and Ala23 were not necessary for myddosome interaction (Figure 3), they are of importance for the interaction with IRAK-1 (Figure 4). Mutation of Tyr105 to alanine did not affect the interaction with IRAK-1. The co-expression of IRAK-1 leads to modification of IRAK-M resulting in an 8 kD MW shift, as previously described by Nechama et al. (2018) to be the result of phosphorylation of Ser110 of IRAK-M and subsequent isomerization of the p-Ser110/Pro111 motif recognized by Pin1. Modification of IRAK-M by IRAK-1 appears to be dependent on the direct interaction of the two proteins since IRAK-1 induced modification of IRAK-M is virtually absent for both the W74A and R97Q IRAK-M mutants.

### 2.3 The IRAK-M type Ia binding surface containing Lys60 is required for a stable IRAK-M homotetramer that drives NF-κB activation in 293T cells

**FIGURE 5:**
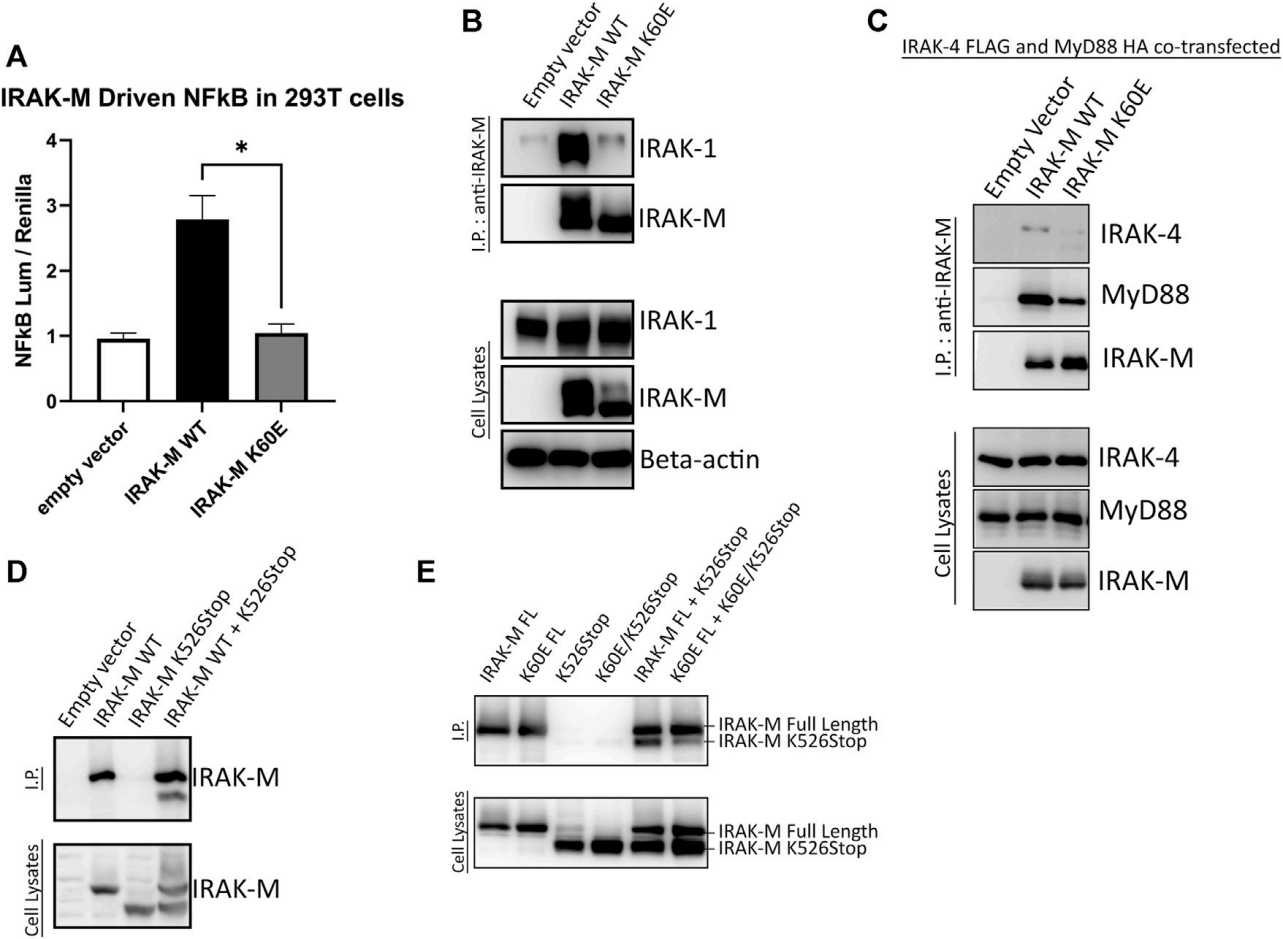
IRAK-M-K60E substitution destroys IRAK-M homo-tetramerization, endogenous capacity of IRAK-M to activate NF-κB and IRAK-1 interaction. **(A)** Effect of IRAK-M K60E mutation on NF-κB activation by overexpression in 293T cells. N = 4, error bars represent mean ± SEM, *p < 0.05. **(B)** IRAK-M WT and K60E mutant were coexpressed with IRAK-1 in 293T cells and subsequently immunoprecipitated using anti-IRAK-M (1F6) antibody. IRAK-1 and IRAK-M were detected by immunoblotting using anti-IRAK-1 and anti-IRAK-M (4369) antibodies. β-actin is used as loading control. **(C)** IRAK-M-WT and K60E IRAK-M mutant were coexpressed with IRAK-4-FLAG and MyD88-HA in 293T cells and subsequently immunoprecipitated. Immunoprecipitation was performed using anti-IRAK-M (1F6) antibody. IRAK-4 and MyD88 were detected using anti-FLAG and anti-HA antibodies consecutively. IRAK-M was detected using anti-IRAK-M (4369). β-actin is used as loading control. All data is representative of three independent experiments. **(D)** IRAK-M WT and IRAK-M K526Stop mutants were expressed in 293T cells and immunoprecipitated using anti-IRAK-M (1F6) antibody, which recognizes the end of the C-terminal Domain. IRAK-M was detected using another IRAK-M antibody that targets total IRAK-M (A5467). **(E)** A combined K60E/526 stop mutant was co-expressed with the full-length K60E IRAK-M mutant and immunoprecipitation with 1F6 and immunoblotting was performed as in **(D)**.

Above we investigated the importance of residues Trp74 and Arg97 for the functioning of IRAK-M assuming that these residues are located on opposite sides of IRAK-M DD homotetramers. In order to determine the importance of IRAK-M DD mediated tetramer formation for the different functions of IRAK-M we generated an IRAK-M mutant, K60E, that lacks the pivotal residue involved in the type Ia interaction of the IRAK-M DD (see Figure 1C) needed to form tetramers as predicted by Du et al. (2014). IRAK-M mutant K60E lacks the ability to activate NF-κB when overexpressed in 293T cells (Figure 5A), indicating that the endogenous capacity of IRAK-M to activate NF-κB depends on DD tetramerization. The IRAK-M/IRAK-1 complex (formed in the absence of myddosome) is also disrupted by the K60E substitution (Figure 5B). In contrast, the K60E mutant interacted with the MyD88/IRAK-4 complex, although with apparent less affinity compared to WT IRAK-M (Figure 5C). Finally, to show the ability of IRAK-M to self-associate via the DD, we generated another IRAK-M mutant (K526Stop), lacking the end part of C-terminal domain and coexpressed with IRAK-M WT. When immunoprecipitated with an anti-IRAK-M antibody targeting the end of C-Terminal Domain IRAK-M K526Stop was immunoprecipitated when coexpressed with IRAK-M, but not by itself (Figure 5D), supporting IRAK-M’s ability to self-associate. Indeed a co-expressed combined K60E/526stop mutant shows considerably less interaction with immuno-precipitated full-length K60E mutant than 526stop with full-length WT IRAK-M (Figure 5E), indicating that the type Ia interaction of the IRAK-M DD is indeed very important for self-association of IRAK-M.

Overall, our findings indicate that the type Ia homotypic interactions of IRAK-M’s DD facilitate homo-oligomerization of IRAK-M in solution at higher concentrations which then allows stable IRAK-1 binding to IRAK-M through the type II interactions provided by Trp74 and Arg97. Furthermore, bound to the MyD88/IRAK-4 scaffold, the type Ia interactions of the IRAK-M DDs appear to stabilize the W74 mediated IRAK-M binding in the myddosome by increased avidity of the IRAK-M homotetramer to the MyD88/IRAK-4 complex.

### 2.4 Direct IRAK-M mediated NF-κB activation does not depend on other IRAK family members

**FIGURE 6:**
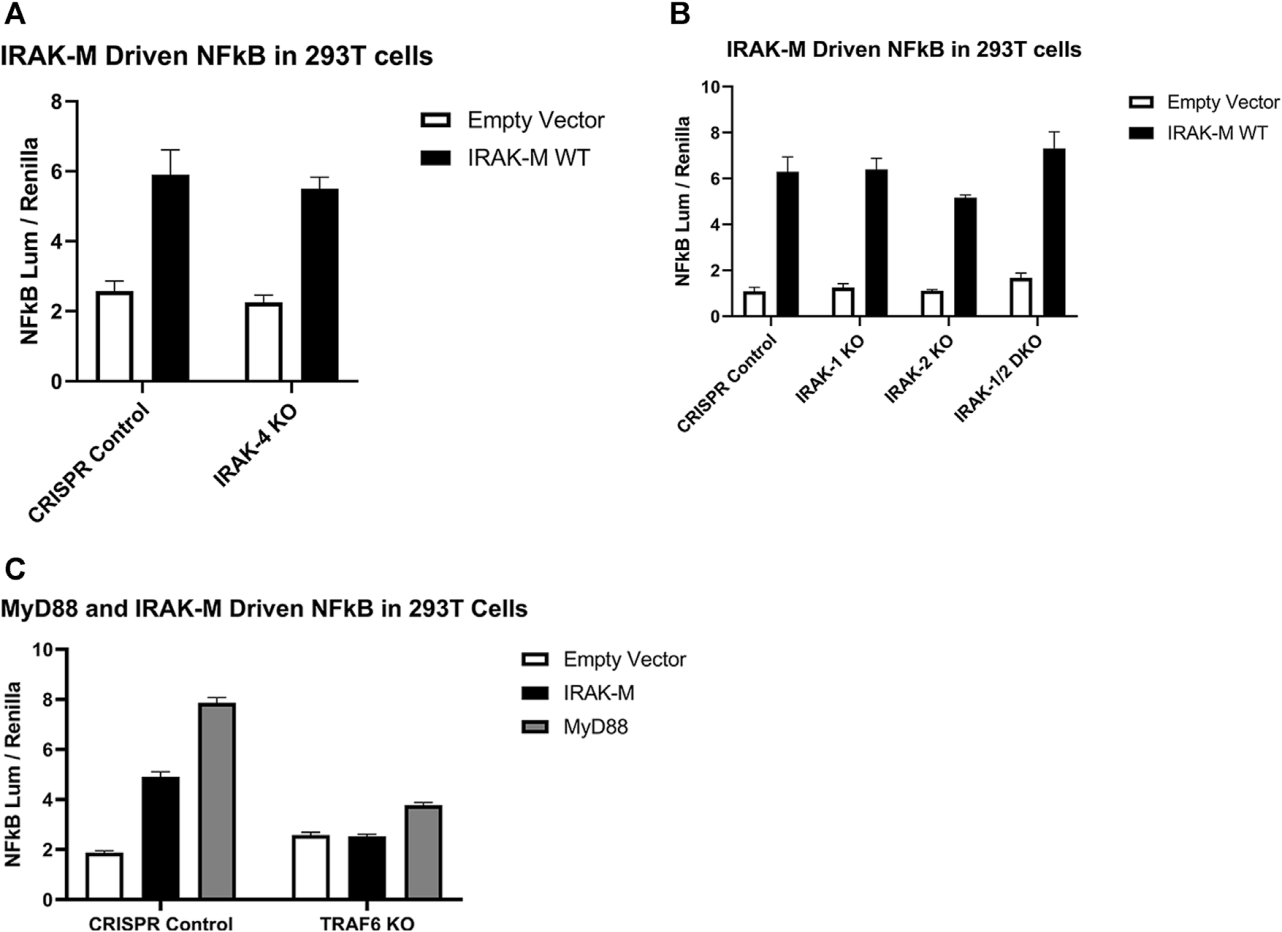
Endogenous capacity of IRAK-M to activate NF-κB is independent of other IRAK’s but depends on TRAF6. IRAK-M-WT driven NF-κB activation by overexpression (200 ng/well) in **(A)** IRAK-4-KO, **(B)** IRAK-1-KO, IRAK-2-KO, IRAK-1/IRAK-2 Double KO and **(C)** TRAF6-KO 293T cells. N = 4, error bars represent mean ± SEM. Data is representative of 3 independent experiments.

In order to determine whether NF-κB activation by bold overexpression of IRAK-M occurs by IRAK-M homo oligomers or by hetero oligomerization with other IRAKs, we generated IRAK-1, IRAK-2 and IRAK-4 knockout 293T cells, as well as IRAK-1/IRAK-2 double knockout 293T cells, and over expressed IRAK-M in these cells to evaluate NF-κB activation. IRAK-M still activated NF-κB in both single and double knockout cells (Figures 6A, B), whereas, as expected, MyD88 overexpression could not activate NF-κB in cells lacking IRAK-1 or IRAK-4, but activated NF-κB to the same level as the control cells in IRAK-2 KO cells (Supplementary Figure S2). This finding is consistent with the notion that IRAK-1, rather than IRAK-2, is the dominant IRAK family member for MyD88/IRAK-4 mediated NF-κB signaling in human cells, (Sun et al., 2016). In agreement, even upon overexpression of both human IRAK-M and IRAK-2 we were unable to detect IRAK-M/IRAK-2 binding (data not shown). Since NF-κB activation by IRAK-M overexpression was not altered by the absence of other IRAKs, we conclude that IRAK-M homo oligomers can activate NF-κB independent of other IRAK family members. This independent activation of NF-κB by IRAK-M overexpression in 293T cells is dependent on TRAF6 as shown by the lack of NF-κB activation by WT IRAK-M in TRAF6 knockout cells (Figure 6C).

### 2.5 IRAK-M binding to TRAF6

**FIGURE 7:**
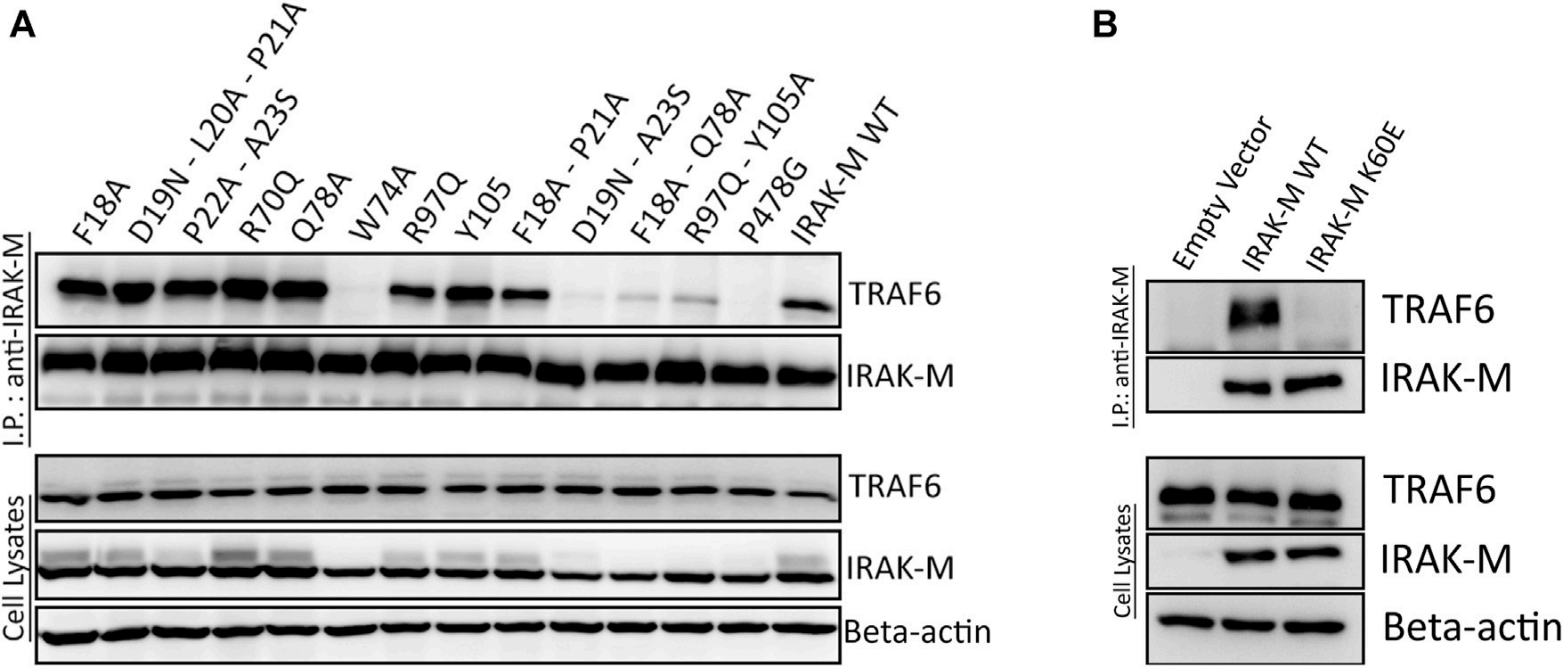
IRAK-M/TRAF6 interaction is mediated by IRAK-M DD residues Trp74 and Arg97/Tyr105. **(A,B)** IRAK-M-WT and indicated IRAK-M mutants were overexpressed in 293T cells and subsequently immunoprecipitated using anti-IRAK-M (1F6) antibody. TRAF6 and IRAK-M were detected using anti-TRAF6 and anti-IRAK-M (4369) antibodies. F18A–P21A stands for the combined mutated stretch “F18A-D19N-L20A-P21A,” D19N–A23S stands for the combined mutated stretch “D19N-L20A-P21A-P22A-A23S.” Data is representative of 3 independent experiments.

IRAK-M tetramers form stable complexes with TRAF6 employing all DD tetramer sides and its C-terminal located TRAF6 binding site independent of other IRAK’s. We previously showed that activation of NF-κB by IRAK-M overexpression is totally dependent on both the Trp74 side as well as the Arg97 side of what we can conclude is a homo oligomer based on the above results observed in otherwise IRAK depleted cells. Upon expression in 293T cells, IRAK-M forms stable complexes with TRAF6, independent of other IRAKs (Supplementary Figure S3) via the single TRAF6 binding motif in IRAK-M pivoted by Pro478, mutation of which abolished TRAF6 binding completely (Supplementary Figure S3; Figure 7A). Stable IRAK-M/TRAF6 complexes also rely on type IIa and type IIb interactions of the DD of IRAK-M since TRAF6 interaction is lost upon mutation of IRAK-M DD residue Trp74 as well as the combined mutation of R97Q/Y105A, although the R97Q and Y105A single mutants still bound TRAF6 to comparable levels as WT IRAK-M (Figure 7A). IRAK-M/TRAF6 interaction depends also on the type Ia DD interaction that forms IRAK-M homotetramers spontaneously upon overexpression as shown by lack of TRAF6 binding of IRAK-M K60E upon overexpression in 293T cells (Figure 7B). TRAF6 needs to trimerize with its TRAF domain to activate NF-κB (Ye et al., 2002).

### 2.6 Activation of NF-κB by the MyD88/IRAK-4/IRAK-M myddosome requires all three binding sites of IRAK-M DD and its P478 C-terminal TRAF6 binding site

**FIGURE 8:**
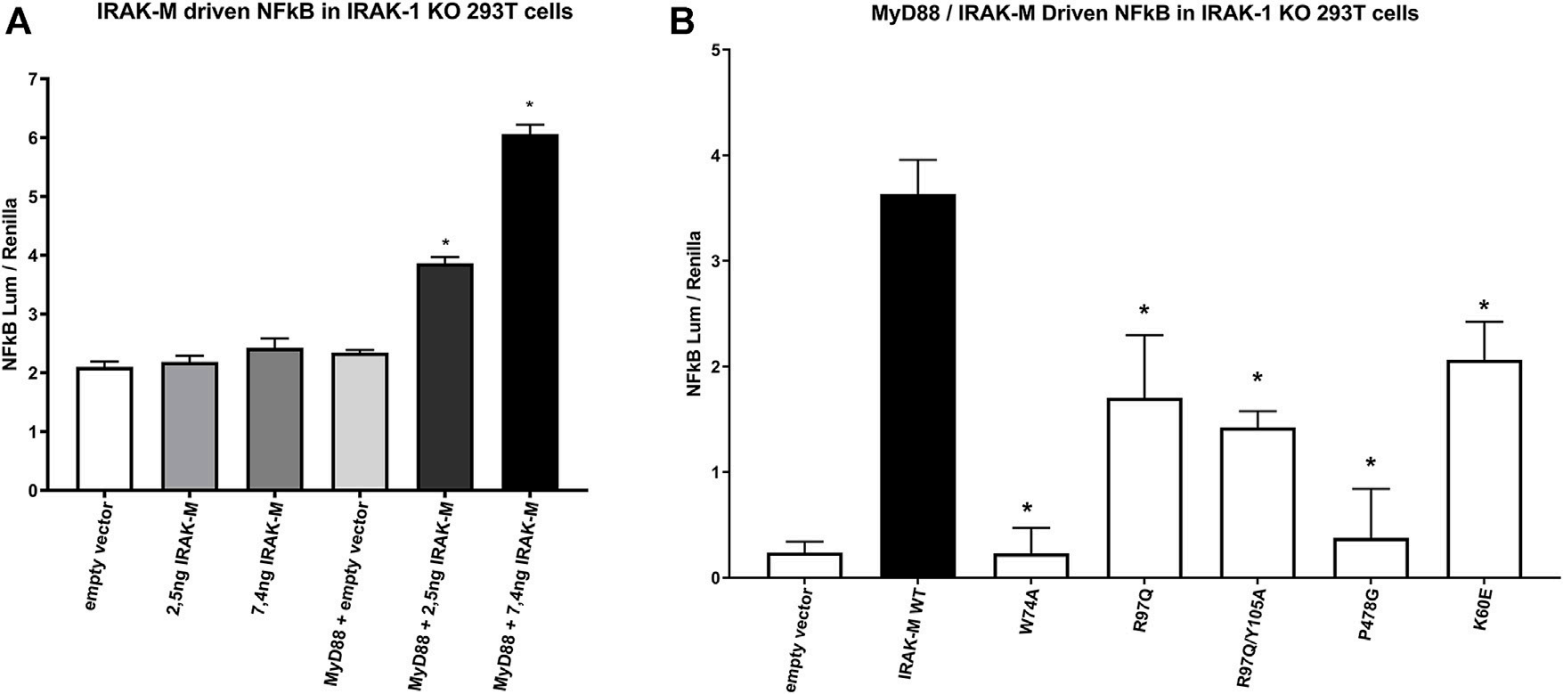
IRAK-M mediated MyD88 driven NF-κB activation involves all 3 sides of IRAK-M’s DD and its own TRAF6 binding site. **(A)** IRAK-M WT was transfected at low levels (2.5 and 7.4 ng/well) in IRAK-1 KO 293T cells with or without the coexpression of MyD88 to induce NF-κB activation. N = 4, error bars represent mean ± SEM, *p < 0.05 compared to MyD88/empty vector coexpression. Data is representative of 3 independent experiments. **(B)** IRAK-M WT and indicated IRAK-M mutants were transfected at low level (7.4 ng/well) in IRAK-1 KO 293T cells together with MyD88 (66 ng/well) to induce IRAK-M/myddosome dependent NF-κB activation. Mean NF-κB levels measured for IRAK-M WT and the individual mutants without MyD88 cotransfection were subtracted. N = 4, error bars represent mean ± SEM, *p < 0.05 compared to IRAK-M WT. Data is representative of three independent experiments.

As shown above, NF-κB activation directly by IRAK-M overexpression in 293T cells is not dependent on the presence of other IRAK family members (Figure 6), but does depend on the formation of IRAK-M homo oligomers which require the type Ia Lys60 dependent interface. To investigate the requirements of the IRAK-M myddosome to activate NF-κB we used IRAK-1 knockout cells. Previous studies showed that MyD88 induced IRAK-M dependent NF-κB activation can be studied in IRAK-1 knockout 293T cells (Li et al., 2001; Zhou et al., 2013). Indeed, MyD88 driven NF-κB activation in 293T cells is absent in IRAK-1 KO cells (Supplementary Figure S2), and already at low transfection levels IRAK-M can substitute for IRAK-1 to restore NF-κB activation via MyD88 triggering in these cells (Figure 8A). Using these IRAK-1 KO cells and low transfection levels we tested the ability of different IRAK-M mutants to act as mediators of NF-κB activation by the MyD88/IRAK-4 myddosome. In line with the lack of interaction of the W74A mutant to the MyD88/IRAK-4 complex (18, Figures 2, 3) the W74A mutant is deficient in generating MyD88/IRAK-4 dependent NF-κB (Figure 8B). The R97Q and R97Q/Y105A mutant activated NF-κB, but with 50% reduced efficiency compared to WT (Figure 8B). A similar 50% reduction in Myd88/IRAK-4 driven NF-κB activation was observed for the K60E mutant that is deficient in the type Ia binding of IRAK-M DDs to form homotetramers (Figure 8B). Furthermore, the MyD88/IRAK-4 dependent NF-κB activating activity of IRAK-M is significantly dependent on its own C-terminal TRAF6 binding motif at Pro478 since the P478G mutant is critically hampered in this function (Figure 8B). These observations indicate that IRAK-M employs all three of its interaction sites on the Death Domain to efficiently drive the MyD88/IRAK-4 mediated NF-κB activation via its own C-terminal TRAF6 binding site.

### 2.7 MyD88/IRAK-4/IRAK-M mediated NF-κB activation does not involve Arg97/MEKK3 interaction

**FIGURE 9:**
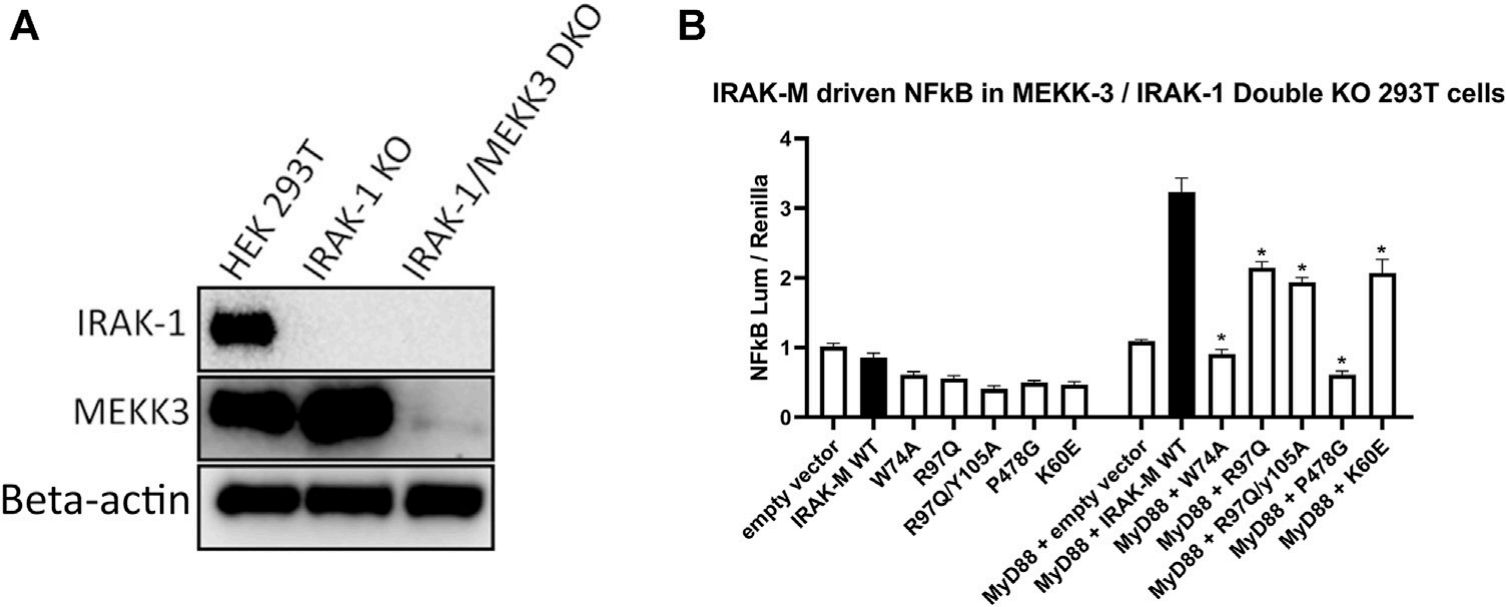
IRAK-M mediated MyD88 driven NF-κB activation does not involve MEKK3 activation via an IRAK-M residue Arg97 dependent process. **(A)** Generation of IRAK-1/MEKK3 Double Knockout 293T cells. IRAK-1 and MEKK3 were detected by immunoblotting using anti-IRAK-M and anti-MEKK3 antibodies. β-actin is used as loading control. **(B)** IRAK-M WT and indicated IRAK-M mutants were transfected at a low level (7.4 ng/well) in the IRAK-1/MEKK3 Double KO 293T cells shown in **(A)** and with or without MyD88 plasmid (66 ng/well) to induce IRAK-M/myddosome dependent NF-κB activation. N = 4, error bars represent mean ± SEM, *p < 0.05. Data is representative of three independent experiments.

IRAK-M driven NF-κB has been described by Zhou et al. (2013) to utilize a MEKK3 dependent pathway in TLR signaling. This prompted us to investigate whether IRAK-M’s residue Arg97 is involved in an interaction with MEKK3 or in MEKK3 dependent NF-κB activation by IRAK-M. At first, we overexpressed MyD88/IRAK-4/IRAK-M together with MEKK3 in 293T cells but we were unable to observe a specific interaction between IRAK-M and MEKK3 in immunoprecipitation studies (data not shown). To test whether MEKK3 is crucial for IRAK-M`s ability to activate NF-κB following myddosome formation we expressed low amounts of IRAK-M in IRAK-1/MEKK3 double KO 293T cells generated for this purpose (Figure 9A). MyD88/IRAK-M coexpression in these IRAK-1/MEKK3 dKO cells still activated NF-κB (Figure 9B), indicating that MEKK3 is not required for IRAK-M mediated MyD88 driven NF-κB activity. Furthermore, also in these IRAK-1/MEKK3 dKO cells, Arg97 was responsible for ∼50% of the IRAK-M dependent MyD88 driven NF-κB activation (Figure 9B), similar to the observation in MEKK3 bearing cells (Figure 8B). This suggests that the Arg97 surface plays a role downstream the myddosome independent of an IRAK-M/MEKK3 interaction.

### 2.8 Structural model for myddosome and homo tetramer formation of the human IRAK-M DD

**FIGURE 10:**
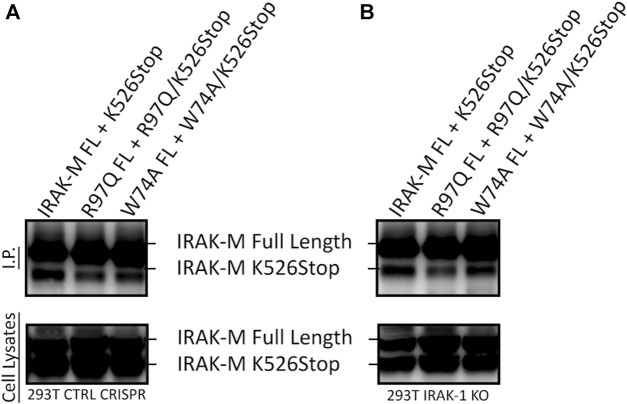
IRAK-M self-association also involves R97. **(A)** Full length IRAK-M WT and the R97Q and W74A mutants were co-expressed with their respective 526stop mutant as in Fig.5E in CRISPR control 293T cells and immunoprecipitated using anti-IRAK-M (1F6) antibody, which recognizes the end of the C-terminal Domain. IRAK-M was detected using another IRAK-M antibody that targets total IRAK-M (A5467). **(B)** As in **(A)** but IRAK-M and mutants were co-expressed in IRAK-1 KO 293T cells.

In contrast to Lys60, Arg97 is not involved in tetramerization of the IRAK-M DD, nor in binding to IRAK-4, but is involved in MyD88/IRAK-4/IRAK-M driven NF-κB in IRAK-1 deficient cells as well as in IRAK-1/MEKK3 double deficient cells (Figures 8, 9). Thus, the contribution of Arg97 is independent of MEKK3. Because our other data indicates that IRAK-M activates NF-κB by homo oligomerization, this prompted us to investigate whether the IRAK-M DD tetramer could be further extended into a homo-pentamer and beyond to an IRAK-M homo-octamer. The ability of R97 to contribute to the self-association of IRAK-M as shown by co-IP of 526stop mutants with full-length IRAK-M (Figures 5D, E) was investigated by co-expression of a combined R97Q/526stop mutant with the full-length R97Q IRAK-M mutant. The R97Q/526stop mutant displayed less interaction with immuno-precipitated full-length R97Q mutant than 526stop with full-length WT IRAK-M (Figure 10A), indicating that the type IIb interaction of the IRAK-M DD is also involved in self-association of IRAK-M. Similar co-IP of a combined W74A/526stop mutant with the full-length W74A mutant displayed a less important role of W74 in self-association of IRAK-M (Figure 10A). The reduced co-IP of R97Q/526stop with full-length R97Q compared to 526stop with full-length WT IRAK-M was also observed in IRAK-1 KO cells (Figure 10B), in line with the notion that this is self-association of IRAK-M.

**FIGURE 11:**
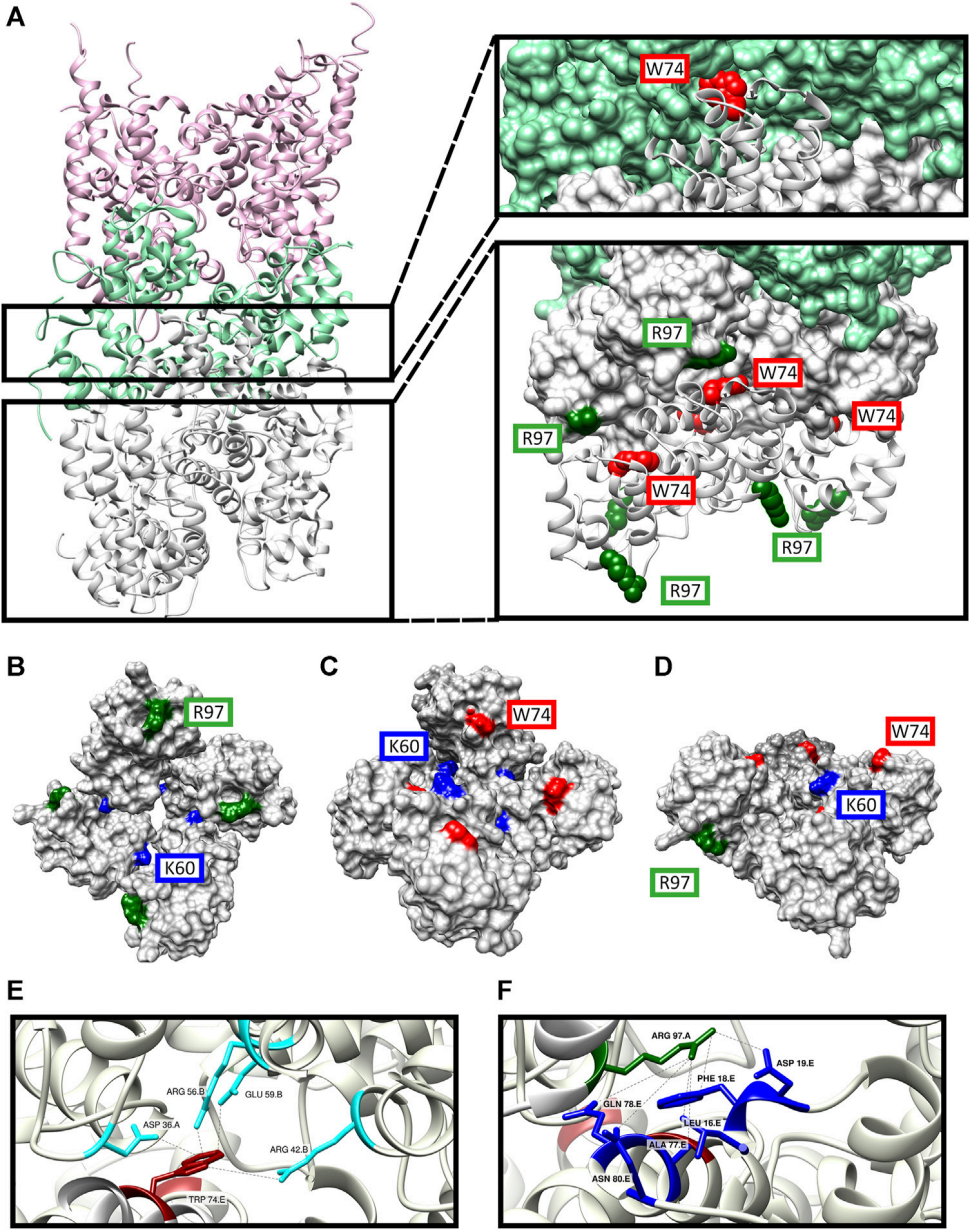
Structure model of the DD’s of MyD88/IRAK-4/IRAK-M myddosome with potential IRAK-M octamer formation via its Arg97 residue. **(A)** The model consists of 6 MyD88 (pink), 4 IRAK-4 (light green) and 8 IRAK-M (grey) molecules in a left-handed helix assembly. IRAK-M/IRAK-4 interaction is pivoted by Tyr74 residue on IRAK-M DD (red). IRAK-M/IRAK-M interaction is pivoted by the Arg97 (dark green) of the top tetramer and the Trp74 (red) of the bottom tetramer. **(B–D)** Proposed IRAK-M tetramer model that forms independent from the myddosome from the top **(B)**, bottom **(C)** and side **(D)** views. Trp74 (red) and Arg97 (dark green) are present on the opposite sides of the tetramer providing the DD interactions reported herein. Lys60 (blue) residues are present in between the individual IRAK-M molecules helping form the tetramer. **(E)** Homo-interactions of IRAK-M residue Trp74 (red) in the assembled IRAK-M octamer helix shown in the right lower panel in **(A)**. **(F)** Homo-interactions of IRAK-M residue Arg97 (dark green) in the assembled IRAK-M octamer helix shown in the right lower panel in **(A)**. Letters behind the labeled residues in panel E and F indicate the different IRAK-M momoner unit they are part of.

To substantiate our findings with structural information we performed a structural superposition of our recently published IRAK-DD homology model (Poelman et al., 2022) to each of the IRAK-4 and IRAK-2 subunits of PDBId:3MOP to obtain an octamer, assuming conservation of the three-dimensional orientation of DD complexes throughout the different DD organization modes. We then aligned the first four subunits of this octamer to the IRAK-4 layer of PDB ID:3MOP, so that the other four octamer subunits were positioned under this layer (Figure 11A)

**FIGURE 12:**
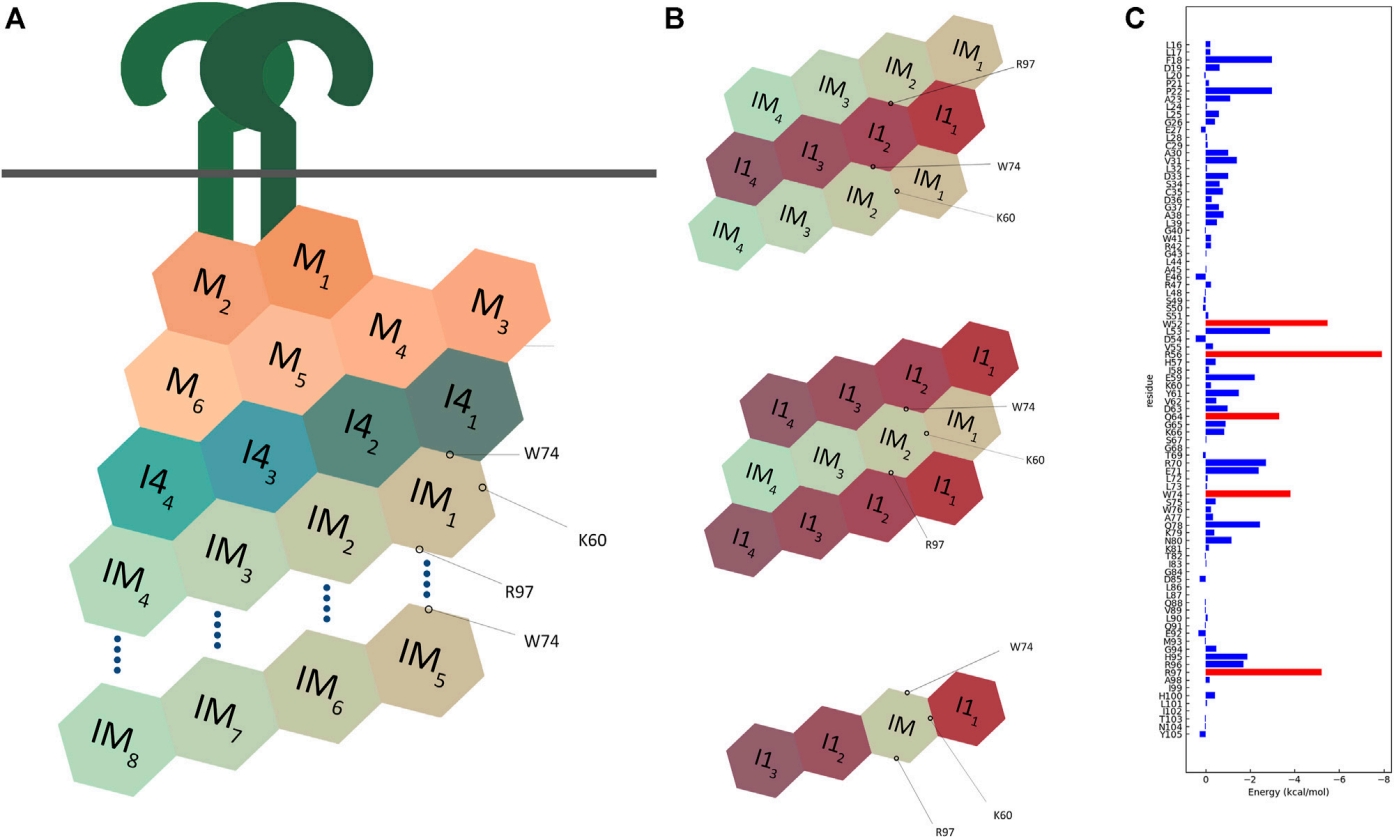
Schematic representation of multimodel interactions of IRAK-M. **(A)** An IRAK-M tetramer layer binds to the IRAK-4 tetramer layer on the formed MyD88/IRAK-4 complex via the type II interactive surface pivoted by Trp74. The Arg97 surface is available for the predicted interaction of a second IRAK-M tetramer layer to bind and form an octamer below the scaffold. Lys60 pivoted surface is responsible for the tetramerization. M = MyD88, I4 = IRAK-4, IM = IRAK-M. **(B)** Two different proposed models for IRAK-1/IRAK-M interaction. Both models use two opposing surfaces of IRAK-M tetramer (Trp74 and Arg97) to interact with IRAK-1 tetramer. This possibility holds for two structurally similar models one of which consists of two IRAK-1 tetramers sandwiching one IRAK-M tetramer, and another consists of two IRAK-M tetramers sandwiching one IRAK-1 tetramer. IRAK-M tetramerization is crucial for both its interactive capabilities and molecular function. This tetramerization occurs through the Lys60 pivoted third interactive surface of IRAK-M but is not regulated by the other two surfaces. A third model that was suggested by Nechama (Nechama et al., 2018) consists of a single IRAK-M monomer replacing one IRAK-1 monomer in an IRAK-1 tetramer. **(C)** Molecular dynamics data of the interactions of the IRAK-M DD residues within the IRAK-M DD homo octamer interactive model (Figure 11), so in between the DD’s IM1-IM8 in Figure 12A. Red bars represent the lowest energies, showing Trp74, Arg97, Trp52, Arg56 and Gln64 as the top interactive residues in the IRAK-M DD octamer.

In this model, the Trp74 of each of the IRAK-M subunits 5 to 8 is positioned on the interface between protomers that are 3 (n−3) and 4 (n−4) up in the helix (Figures 11A, C, E, 12A). Arg97 of each of the IRAK-M subunits 1 to 4 is positioned to allow interaction with Asp19 of subunits that are 4 (n + 4) positions below it in the helix (Figures 11A, B, F, 12A). Lys60 of the subunits is positioned to interact with subunit n−1 (Figures 11B, C, 12A), possibly with Asp33 although this residue is also involved in a conserved intramolecular hydrogen bond (Poelman et al., 2022). The position of IRAK-M residue Tyr105 is not well conserved in the death domain family but most probably positioned peripherally on the octamer without interacting points with the other IRAK-M DD subunits (Supplementary Figure S4).

We used SCRWL4 (Krivov et al., 2009) to debump the sidechains of the octamer and ran a molecular dynamics (MD) simulation with the debumped octamer, followed by energy decomposition per residue (Figure 12C). As expected, Trp74 and Arg97 showed good binding energies. Other residues that gave good interaction energies were Trp52 and Arg56, which in the octamer model are located on the interface between n and n + 3 and n and n−1 respectively. This energy decomposition suggests that Lys60 hardly contributes to the Ia interaction, and it appears that the K60E substitution in the mutant used herein flips the Ia interaction towards unfavorable.

## 3 Discussion

Here we describe how human IRAK-M uses three distinct surface areas on its Death Domain to form homo- and hetero-multimers with proteins in the MyD88 driven TLR/IL-1 receptor signaling pathway and thereby provide IRAK-M the capacity to interact through multiple modes. In otherwise IRAK deficient cells we show that the type Ia interaction, as defined by Lin et al. (2010) for the MyD88/IRAK DD interactions, and in IRAK-M the surface around central Lys60, enables formation and stability of predicted IRAK-M homotetramers that can bind TRAF6 and activate NF-κB in a homotypic manner (Figures 5, 6). One of the sides of the IRAK-M homotetramer exposes the interactive IIa face containing the Trp74 residue of 4 DD’s. This Trp74 interactive face is essential for the interaction of IRAK-M with IRAK-4 in the myddosome scaffold (MyD88/IRAK4) as previously reported (Zhou et al., 2013) and confirmed herein. The extended surface and valency provided by the type Ia interaction (see Figures 11, 12) appear to stabilize the interaction of IRAK-M with the myddosome, since myddosome binding (Figure 5C) and MyD88/IRAK-4/IRAK-M-induced NF-κB (Figure 8B) were reduced by 50% upon K60E substitution. This while the K60E mutant completely lost the capacity to activate NF-κB (Figure 5A) and TRAF6 interaction (Figure 7B) upon expression in the absence of the myddosome scaffold. Most likely binding of the K60E mutant to the myddosome tetramer, although reduced, brings again enough TRAF6 binding sites together to generate NF-κB. In the MD analysis of the IRAK-M DD octamer, Lys60 does not appear an important contributor for the Ia interaction, the change from lysine to glutamic acid presumably results in destruction of the type Ia interaction by electrostatic repelling of Glu60 and Asp33. The third interactive face IIb of the IRAK-M DD, opposite of Trp74, and exposed on the other side of the homotetramer contains residue Arg97. This Arg97 face is not involved in IRAK-M binding to the MyD88/IRAK-4 scaffold (Figures 2, 3) in full agreement with the predicted location of the four Arg97 residues in an IRAK-M homotetramer (Figure 11). Remarkably, mutation of Arg97 reduced MyD88/IRAK-4/IRAK-M induced NF-κB activation (Figure 8B). This was observed in IRAK-1 deficient cells, indicating that another protein or IRAK-M itself may provide enhancement of the NF-κB signal via an Arg97 dependent interaction. In agreement, we previously showed that R97Q substitution reduced the capacity of IRAK-M to activate NF-κB upon over-expression (Du et al., 2014), here shown to involve homotypic interactions (Figure 6), by 80% and even by 100% in combination with the Y105A substitution (Du et al., 2014). Moreover, here we show that Arg97 in combination with Tyr105 is important for the binding of TRAF6 to an IRAK-M homo multimer, an interaction that also involves Trp74. To activate NF-κB TRAF6 needs to trimerize (Ye et al., 2002), Since IRAK-M has only 1 TRAF6 binding site in the C-terminal stretch our findings prompt the question how the different molecules in the hypothesized IRAK-M octamer orient their C-terminal tail to accommodate TRAF6 trimerization.

Zhou et al. (2013) described the interaction between IRAK-M and MEKK3 in murine cells by immunoprecipitation assay. We did however not observe MEKK3 interaction with IRAK-M in immunoprecipitation experiments with the human myddosome proteins overexpressed. Furthermore, MyD88 mediated NF-κB activation was still induced by IRAK-M in IRAK-1/MEKK3 double knockout cells, wherein the R97Q mutant showed a significant reduction in NF-κB compared to WT IRAK-M. These observations indicate that it is highly unlikely that the features of IRAK-M mediated by its Arg97 residue are the result of a MEKK3 dependent event. These findings suggest other pathways for IRAK-M mediated NF-κB activation then the MEKK3 pathway. Further studies are required to investigate which other mediator(s) are involved in IRAK-M’s capacity to activate NF-κB. Upon co-expression IRAK-M stably interacts with IRAK-1 (Figure 4). Remarkably this myddosome independent interaction with IRAK-1 was dependent on the Lys60, Trp74 and Arg97 interactive faces of the IRAK-M DD (Figures 4, 5). This suggests that both the IIa as well as the IIb side of IRAK-M DD homotetramers are engaged in IRAK-1 binding. If IRAK-M homotetramerization precedes IRAK-1 binding this would involve a novel mechanism for IRAK-M/IRAK-1 interaction where an IRAK-1 tetramer is sandwiched by two IRAK-M tetramers via their Trp74 and Arg97 faces of DD, or *vice versa* where one IRAK-M tetramer is sandwiched by two IRAK-1 tetramers again using both sides of IRAK-M DD for a stable interaction (Figure 12B). Alternatively, IRAK-M could form heterotetramers with IRAK-1 as suggested by Nechama et al. (2018) based on structure modeling studies of IRAK-M binding to a MyD88/IRAK-4/IRAK-1 complex. Nechama and coworkers predicted that a Tyr105/Leu20-Pro21 interaction within the IRAK-M DD renders an IRAK-M DD conformation that supports IRAK-1 binding and that this Tyr105/Leu20-Pro21 interaction is released by the isomerization of the p-Ser110/Pro111 motif resulting in dissociation of IRAK-1 (Nechama et al., 2018). However, here we found that alanine substitution of IRAK-M residue Tyr105 did not alter IRAK-1 interaction, an observation that does not support the involvement of Tyr105 in the interaction between IRAK-M and IRAK-1. In this study, we employed a combination of co-immunoprecipitation assays, functional analyses, and computational modeling to elucidate the structural and functional dynamics of IRAK-M DD and it’s interaction with other molecules. While these methods have enabled us to generate robust predictions regarding the structure function of IRAK-M DD, it is clear that further experiments, such as X-ray crystallography, are necessary to confirm the octamer model as proposed in Figure 11. The purification and structure of MyD88, IRAK-2 and IRAK-4 DDs has been published (Lin et al., 2010). The expression of isolated IRAK-M and IRAK-1 DDs and purification thereof is however frustrated by the low yield and precipitation of WT IRAK-M DD’s (29, and own observation) and instability of WT IRAK-1 DD’s (Lin et al., 2010). According to an siRNA-based screening it was revealed that IRAK-2 is the dominant activator of the pathway in murine cells while IRAK-1 is dominant in human cells (Sun et al., 2016). As our study is performed with human cells and proteins, we are basing our findings on a myddosome scaffold that recruits an IRAK-1 tetramer for signal transduction. Two modes of interaction have been suggested so far for IRAK-M in the MyD88 dependent pathway. The first mode of interaction is where IRAK-M is able to bind the IRAK-1 (human) or IRAK-2 (mouse) tetramer on the myddosome (Kobayashi et al., 2002), inhibiting the dissociation of the tetramer, and thereby inhibiting the signal. In the second mode of interaction IRAK-M binds to the scaffold directly via the IRAK-4 tetramer and transduce the signal via a MEKK3 dependent pathway, activating NF-κB that will induce transcription of certain anti-inflammatory proteins such as A20, IκBα and SOCS1 (mouse) (Zhou et al., 2013). To our knowledge the latter effect has not been shown yet in human monocytes/macrophages or lung epithelial cells.

With regard to the contribution of the Arg97 residue to the inhibitory effect of IRAK-M we previously observed that the R97Q variant of IRAK-M has decreased inhibitory capacity on cytokine release by human monocytes triggered via TLR2 and TLR4, while the R97Q variant has increased capacity to inhibit cytokine release triggered via TLR5 in lung epithelial cells (Du et al., 2014). The main difference between the myddosomes formed by TLR2/4 with myddosomes formed by TLR5 is that TLR2 and TLR4 will mediate long oligomers of MyD88 under the influence of MAL (Ve et al., 2017), while TLR5 is thought to generate the classic MyD88 dimer + tetramer myddosome. With our present knowledge on the location of IRAK-M’s Arg97 residue and importance in IRAK-1 binding it may be hypothesized that the lower capacity of the R79Q mutant to inhibit TLR2/4 cytokine production in monocytes is dependent on a lower capacity to interfere with the long MAL induced MyD88 oligomers and reduced IRAK-1 binding. The higher potency of the R97Q variant to inhibit TLR5 myddosome may be caused by its untouched capacity to bind to the myddosome, but decreased ability to activate NF-κB and cytokine production. In this regard, the capability of IRAK-M to inhibit TLR5 mediated cytokine production in lung epithelial cells was not associated with upregulation of A20 levels, on the contrary we observed lower levels of A20 (Du et al., 2014). The downstream effect of IRAK-M on NF-κB activation incited by specific TLR agonist should be further investigated. Of interest is that the kinase domain of IRAK-M contains a cryptic guanylate cyclase (GC) center capable of generating anti-inflammatory cGMP (Nguyen et al., 2022; Freihat et al., 2019; D'Elia et al., 2013). An IRAK-M mutant devoid of the GC center (R372L) was partially hampered in reconstituting IRAK-M’s inhibitory function in IRAK-M deficient THP-1 cells indicating that the GC center contributes to this function of IRAK-M. In the same reconstitution experiments the W74A and R97A mutant completely lacked inhibitor function in this report (Nguyen et al., 2022), confirming our previous observations (Du et al., 2014) that both Trp74 and Arg97 are vital for the function of IRAK-M. Our present data indicates that both the Trp74 and Arg97 dependent interactions are important for IRAK-M myddosome functions and IRAK-1 binding, and that these enable TRAF6 binding. Furthermore, these DD interactions will also bring together the kinase domains of IRAK-M.

Elucidation of how different surfaces of IRAK-M interact with other proteins is crucial for our understanding of the regulation of TLR signaling by IRAK-M. As an important anti-inflammatory protein in the MyD88 pathway, targeting of IRAK-M is a potential way of boosting the immune system in patients with bacterial infection, immunoparalysis or cancer, since IRAK-M targeting in animal models of these diseases improves outcome (Kobayashi et al., 2002; Deng et al., 2006; Hubbard et al., 2010; Hoogerwerf et al., 2012; van der Windt et al., 2012). By itself the DD of IRAK-M lacks a druggable pocket as potential target for small molecules, our results indicate that IRAK-M activity could be targeted via influencing the multimerization of IRAK-M DD’s (Ivanov et al., 2013). Although IRAK-M is a pseudokinase, Lange et al. (2021) showed that the kinase domain of IRAK-M adapts an active conformation, binds ATP and staurosporin. The ATP binding site of IRAK-M allows for targeting by PROTAC’s (Degorce et al., 2020; Rowley et al., 2022), chimeric bifunctional molecules which bind the target and attract an E3 ligase to ubiquitinate and degrade the target by the proteasome. Although further research is required, the present study elucidates how the death domain of IRAK-M is involved in the association of IRAK-M to its binding partners which will potentially aid in the development of IRAK-M inhibitors to boost innate immunity.

## 4 Materials and methods

### 4.1 Cell culture

293T cells were grown in complete DMEM, supplemented with 10% FBS, 2 mM L-Glutamine and 100 μg/mL Pen/Strep. Cells were maintained at 37°C in a humified incubator with 5% CO_2_.

### 4.2 Transfection and plasmids

Transfections of 293T cells were carried out at 80% confluency using Lipofectamine 2000 according to the manufacturer`s guidelines (Thermo Fisher Scientific). The amount of transfected coding vector differed depending on the assay, however the total amount of transfected vector was kept constant among samples and experiments, using the empty vectors to ensure equal transfection efficiency. Mammalian pUNO expression plasmids for human IRAK-M, IRAK-1 and MyD88-HA were from Invivogen, CMV expression plasmid for IRAK-4-FLAG was from Sino Biological Inc. IRAK-M mutants were generated in the pUNO IRAK-M plasmid as described (Du et al., 2014) using the QuikChange II kit (Agilent), according to the manufacturer`s guidelines. Constructs were subjected to DNA sequencing to confirm the mutations and check of the appropriate CDS.

### 4.3 Lentivirus production and generation of CRISPR knock out cell lines

The CRISPR-Cas9 system was utilized to silence gene expression. For this sgRNAs targeting the genes in question were selected from the human GECKOv2 sgRNA library (Sanjana et al., 2014) and cloned in lentiCRIPSRv2 vectors containing either a puromycin or blasticidin selection markers (Shalem et al., 2014). Targeting lentiviruses were produced in 293T cells via transfection of pLENTI-CRISPR V2 vector containing the gRNA for selected genes, together with packaging vectors pVSV, pMDL and pRES. The virus containing supernatant medium was collected at 72-h, filtered through a 45 µm filter, aliquoted and stored at −80°C until use (Seppen et al., 2002). For gene silencing 293T cells seeded at 1 × 105 per well in 24 well plates were transduced by addition of 200 µL of collected lentivirus supernatant medium mixed with 50 µL of 80 μg/mL polybrene (van Lent et al., 2009). Knock out cells were selected using 1 μg/mL Puromycin or 10 μg/mL Blasticidin depending on the selection marker of the vector. The knockouts were confirmed using Western blotting and/or Fragment Length Analysis when applicable.

**Table:** 

CRISPR guide sequences	
Name	Sequence
MEKK3 Forward	cac​cgG​GTC​TAT​TTG​TGC​TAT​GAC​G
MEKK3 Reverse	aaa​cCG​TCA​TAG​CAC​AAA​TAG​ACC​c
IRAK-1 Forward	cac​cgG​ATC​AAC​CGC​AAC​GCC​CGT​G
IRAK-1 Reverse	aaa​cCA​CGG​GCG​TTG​CGG​TTG​ATC​c
IRAK-2 Forward	cac​cgC​CCT​CTG​TAG​ACG​TCA​GCA​A
IRAK-2 Reverse	aaa​cTT​GCT​GAC​GTC​TAC​AGA​GGG​c
IRAK-4 Forward	cac​cgA​TGG​CAC​CAG​AAG​CTT​TGC​G
IRAK-4 Reverse	aaa​cCG​CAA​AGC​TTC​TGG​TGC​CAT​c
TRAF6 Forward	cac​cGG​AAG​CAG​TGC​AAA​CGC​CAT​G
TRAF6 Reverse	aaa​cCA​TGG​CGT​TTG​CAC​TGC​TTC​c

### 4.4 Confirmation of knockdown in 293T cells by CRISPR guides

Knockdown of MEKK3 and IRAK-1 by the guides used above was confirmed by Western blotting as shown in Figure 9A. Knockdown of TRAF6 is shown by Western blot in Supplementary Figure S5A. IRAK-2 knockdown was examined using the Fragment Length Analysis method on purified DNA of the cells to confirm the genetic deletion. Forward primers containing 6-FAM tag and untagged reverse primers were used to amplify the CRISPR target region. ROX-500 (Thermo Fisher Scientific) was used to as the standard. The result of the genetic deletion of IRAK-2 by the used guides was analyzed using the GENEMAPPER 5.0 software shown in Supplementary Figure S5B. Knockdown of IRAK-4 in 293T cells was shown functionally by the lack of induction of NF-κB by MyD88 overexpression as shown in Supplementary Figure S5C in line with the requirement of IRAK-4 for myddosome dependent NF-κB activation.

### 4.5 NF-κB activation

293T cells seeded in 96-well cell culture plates were co-transfected with IRAK-M wild type or IRAK-M mutant sequences and a Firefly Luciferase NF-κB driven reporter construct (34 ng/well) and a Renilla Luciferase CMV driven construct (0.7 ng/well) (Promega). Total transfected plasmid DNA was kept constant at 200 ng/well complexed with 0.5 µL Lipofectamine 2000. To determine the effect of mutations on the endogeneous capacity of IRAK-M to activate NF-κB IRAK-M was overexpressed by transfection of >60 ng/well IRAK-M plasmid. To determine the effect of mutations on the capacity of IRAK-M to drive MyD88 dependent NF-κB activation 7.4 ng/well IRAK-M was co-transfected with 66 ng/well MyD88-HA plasmid in IRAK-1 knockout cells. Twenty four hours after transfection cells were washed with PBS and lysed to measure Firefly and Renilla Luciferase activities using the Dual-Glo^®^ Luciferase Assay System (Promega) according to the manufacturer’s guidelines.

### 4.6 Co-immunoprecipitation and immunoblotting

For immunoprecipitation of IRAK-M cells were harvested 24 h after transfection, washed with PBS and lysed in Cell Signaling Technology cell lysis buffer supplemented with HaltTM Protease & Phosphatase Inhibitor Cocktail (Thermo Scientific) on ice and sonicated for 5 s at 30% amplitude (Vibra-CellTM ultrasonicator, Sonics & Materials Inc.) and centrifuged at 14.000 g for 10 min at 4°C. Subsequently 1.5 µg of mouse monoclonal anti-IRAK-M antibody 1F6 (Abnova) raised against the C-terminal part (aa 497–596 of IRAK-M) was added to 300 µL supernatant lysate and the samples were incubated for an hour on ice. Then 50 µL of protein A/G PLUS-agarose beads (Santa Cruz Biotechnology) was added and samples were incubated on a roller at 4°C for an hour. Consecutively the beads were centrifuged and washed 3 times using the Cell Signaling Technology cell lysis buffer and bound proteins were eluted with 50 µL of SDS Sample Buffer (60 mM Tris-HCL pH = 6.8, 3% SDS, 10% glycerol, 5% 2-mercaptoethanol, 0.05% Bromophenol Blue) incubated at 95°C for 5 min and stored at −20°C. Western blotting was performed as described (van’t Veer et al., 2007) with the antibodies listed in the table below

**Table:** 

Antibody list			
Antibodies	Company	Code	Use
IRAK-M	Abnova	1F6	Immunoprecipitation
IRAK-M	Cell Signaling Technology	4369	Western blot
IRAK-M	Neobiolab	A5467	Western blot
FLAG	Sigma Aldrich	F7425	Western blot
HA	Thermo Scientific	PA1-985	Western blot
IRAK-1	Cell Signaling Technology	4359	Western blot
TRAF6	Cell Signaling Technology	D21G3	Western blot
MEKK3	BD Biosciences	Clone 40	Immunoprecipitation
MEKK3	Cell Signaling Technology	D36G5	Western blot
β -actin	Cell Signaling Technology	4967	Western blot

### 4.7 IRAK-M death domain modelling

The IRAK-M octamer was constructed by superposition of a previously published homology model for the IRAK-M DD (Poelman et al., 2022) to the IRAK-4 and IRAK-2 chains in PDB ID:3MOP (Lin et al., 2010), using the MOTIF superposition algorithm in WHAT_IF (Vriend, 1990). Four chains of the octamer were then superposed to the IRAK-2 layer of PDB ID:3MOP, such that the octamer was positioned under the IRAK-4 layer of PDB:3MOP. For the molecular dynamics analysis, the octamer model was first debumped using SCRWL4(31) and was subjected to molecular dynamics (MD) simulation by using standard parameters and protocols as recently described (Wichapong et al., 2021; Hrdinova et al., 2022). Briefly, AMBER ff19SB force field was assigned for protein (IRAK-M), and the explicit OPC water model and counter ions (Na+ or Cl−) were then added to a 10 Å radius from the molecular surface of the protein. Prior to running MD simulation, the solvated IRAK-M was relaxed by performing energy minimization. Then, MD simulation was conducted for 100 ns under standard conditions (i.e., temperature at 300 K, pressure at 1 bar, time step of 2 fs with SHAKE constraint). MD snapshots extracted from the last 20 ns (80–100 ns) were utilized for per-residue decomposition (DC) energy analysis and binding free energy (BFE) calculation by application of the molecular mechanics/generalized Born surface area (MM/GBSA) approach using GB model 8 with default parameter settings (Liu et al., 2021). The average of the four lowest energies was used for each residue.

### 4.8 Statistical analyses

Differences between WT and mutant proteins in qPCR, ELISA and NF-κB Luciferase experiments were calculated by Mann-Whitney U test. Values are expressed as mean ± SEM. A *p* < 0.05 was considered to represent a statistically significant difference.

## Data Availability

The original contributions presented in the study are included in the article/Supplementary Material, further inquiries can be directed to the corresponding author.
