# Supplementary material for: The IRAK-M death domain: a tale of three surfaces
Source: Front Mol Biosci. 2024 Jan 10;10:1265455. doi: 10.3389/fmolb.2023.1265455 (PMC10806146; doi:10.3389/fmolb.2023.1265455)
Supplement: Supplementary file 1 [file DataSheet1.docx]

Supplementary Material

# Supplementary Figures


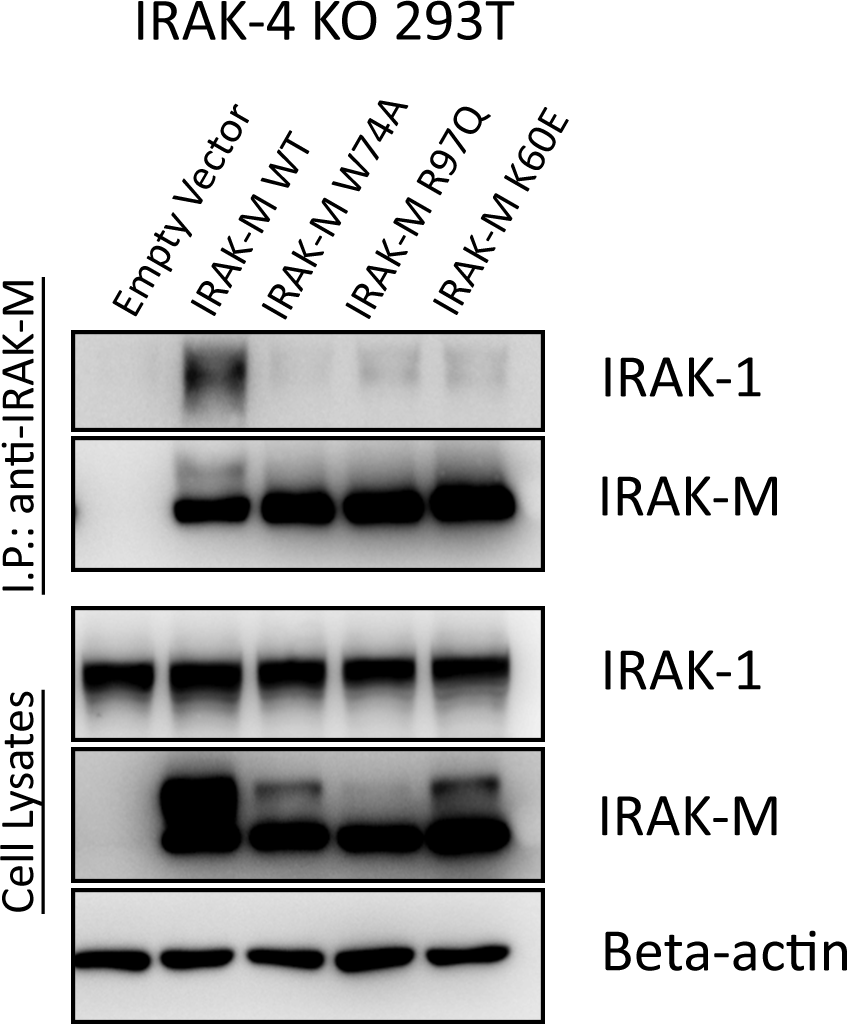


**Supplementary Figure 1.** **IRAK-M/IRAK-1 interaction occurs independent of IRAK-4.** IRAK-M-WT and indicated IRAK-M mutants were cotransfected with IRAK-1 in IRAK-4 KO 293T cells and subsequently immunoprecipitated. Immunoprecipitation was performed using anti-IRAK-M (1F6) antibody. IRAK-1 and IRAK-M were detected using anti-TRAF6 and anti-IRAK-M (4369) antibodies. β-actin is used as loading control.


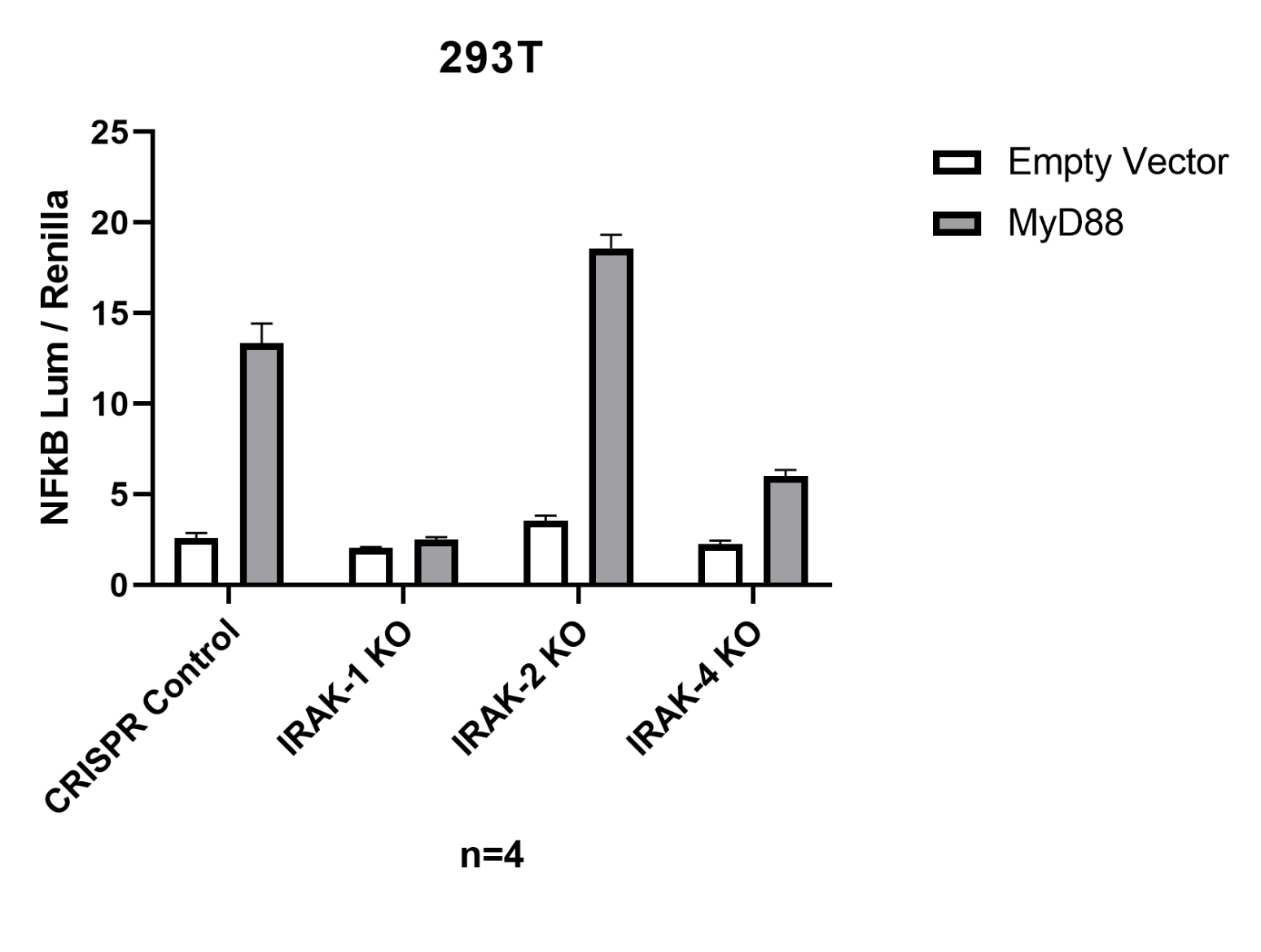


**Supplementary Figure 2. IRAK-1 and IRAK-4 are crucial for MyD88 dependent NF-κB activation in 293T cells.** MyD88-HA was overexpressed in IRAK-1, IRAK-2 and IRAK-4 KO cells to induce myddosome dependent NF-κB activation. N=4, error bars represent mean ± SEM. Data is representative of 3 independent experiments.


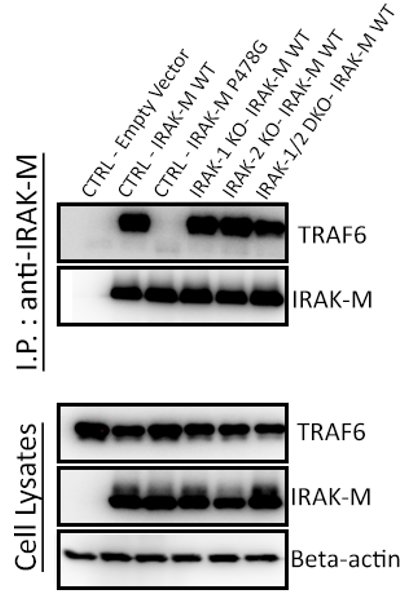


**Supplementary Figure 3. IRAK-M/TRAF6 interaction does not depend on the presence of other TRAF6 binding IRAKs but on its own TRAF6 binding motif.** IRAK-M-WT and IRAK-M P478G mutant were overexpressed in IRAK-1 KO, IRAK-2 KO and IRAK-1/2 DKO 293T cells and subsequently immunoprecipitated. Immunoprecipitation was performed using anti-IRAK-M (1F6) antibody. TRAF6 and IRAK-M were detected using anti-TRAF6 and anti-IRAK-M (4369) antibodies. β- actin is used as loading control.


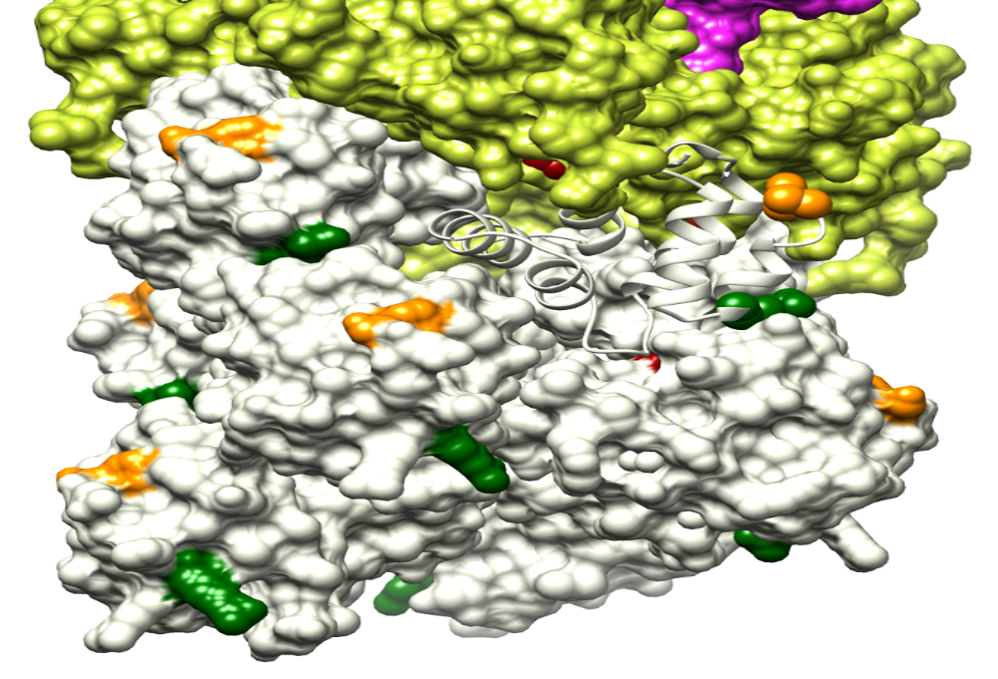


**Supplementary Figure 4.** Position of IRAK-M Tyr105 in the IRAK-M octamer of the modeled MyD88/IRAK-4/IRAK-M shown in Fig.10. Tyr105, Trp74 and Arg97 residues are labeled with orange, red and green respectively.


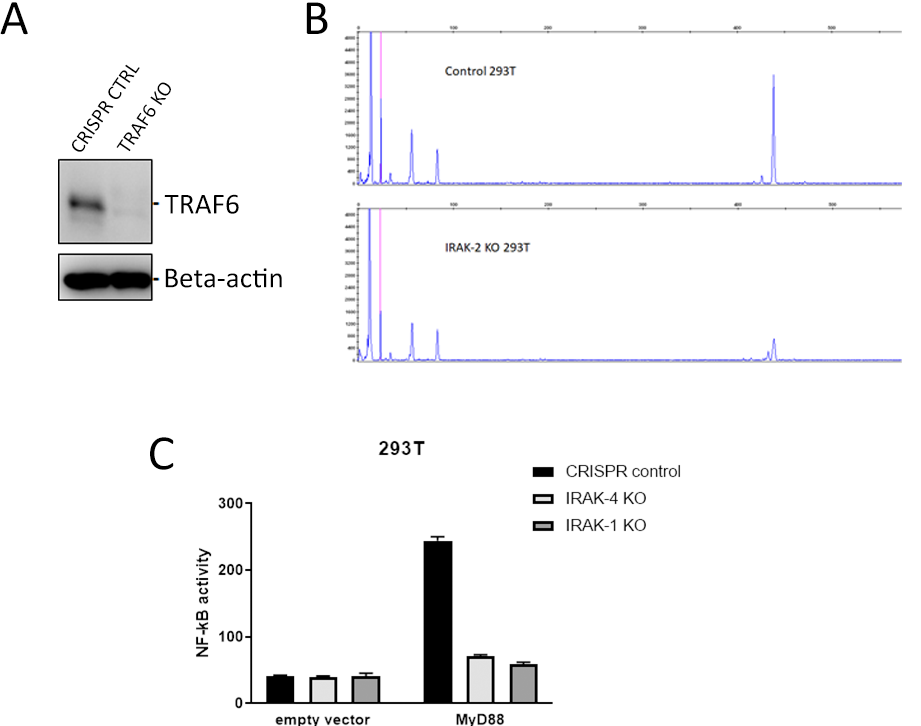


**Supplementary Figure 5.** CRISPR efficiency control in KO cell lines. **A)** Knockdown of TRAF6 shown by western blotting. **B)** Knockdown of IRAK-2 shown by Fragment Length analysis **C)** Knockdown of IRAK-4 shown by the lack of NF-κB activation by MyD88 overexpression.
